# Supplementary material for: Convection enhanced delivery of Rhenium (186Re) Obisbemeda (186RNL) in recurrent glioma: a multicenter, single arm, phase 1 clinical trial
Source: Nat Commun. 2025 Mar 7;16:2079. doi: 10.1038/s41467-025-57263-1 (PMC11889265; doi:10.1038/s41467-025-57263-1)

## SUPPLEMENTARY INFORMATION

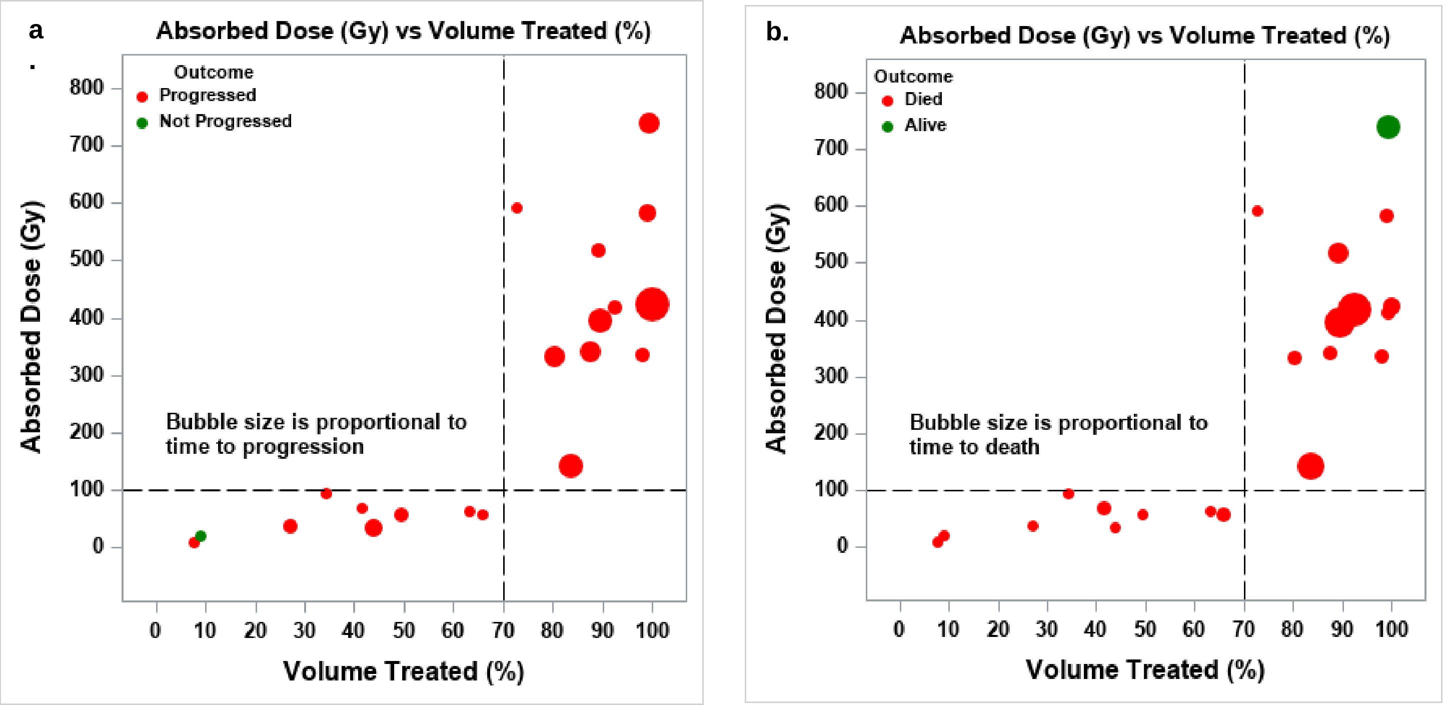


**Supplementary Figures 1a-b. Absorbed dose versus volume treated (%).** 1a. The bubble size is proportional to time to progression. 1b. The bubble size is proportional to time to death. At the time of reporting, one patient remained alive.

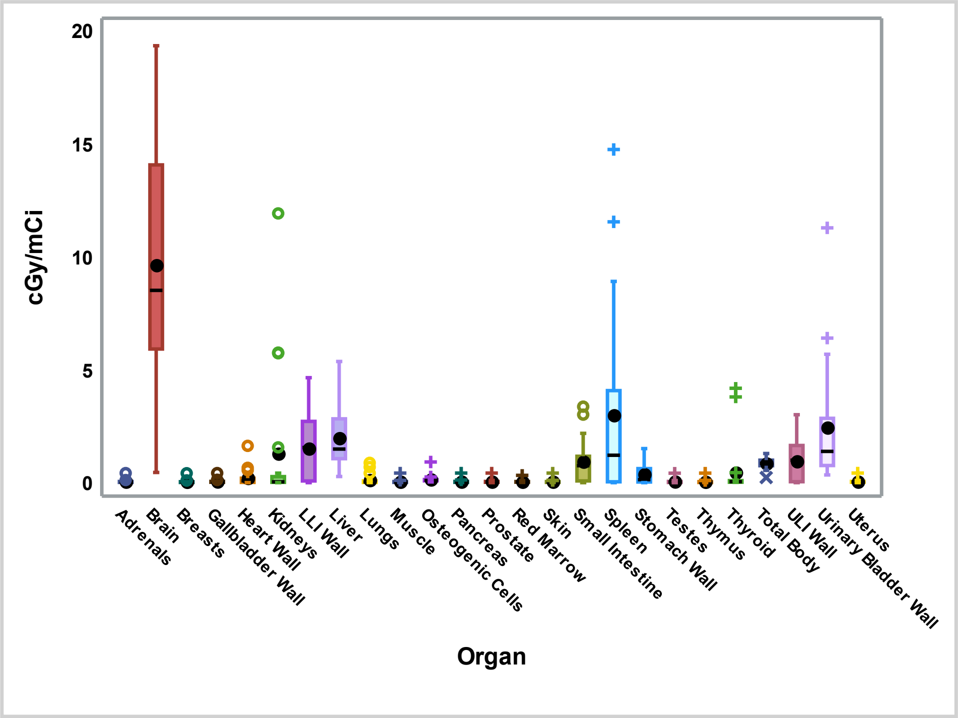


**Supplementary Figure 2. Box and Whisker plot of normalized radiation absorbed dose (cGy/mCi) to organs (N=21).** Brain absorbed dose includes absorbed dose to tumor.

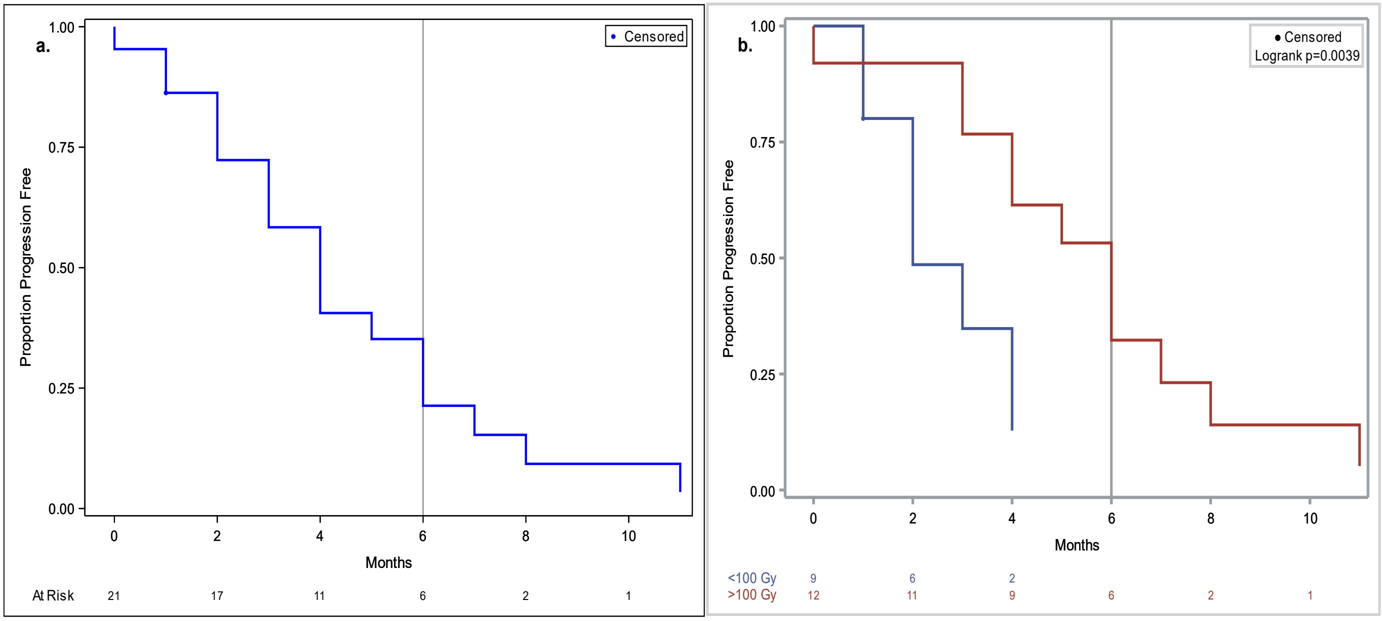


**Supplementary Figures 3a-b. Kaplan-Meier of progression free survival.** 3a. For all patients, the mPFS was 4.0 m (95% CI 2.0-6.0 m, PFS6=0.21±0.11). 3b. When dichotomized by absorbed dose, patients who received <100 Gy had a mPFS of 2.0 m (95% CI 1.0-4.0 m, PFS6=0.0) and those with ≥100 Gy hand a mPFS of 6.0 m (95% CI 3.0-8.0 m, PFS6=0.32±0.16).

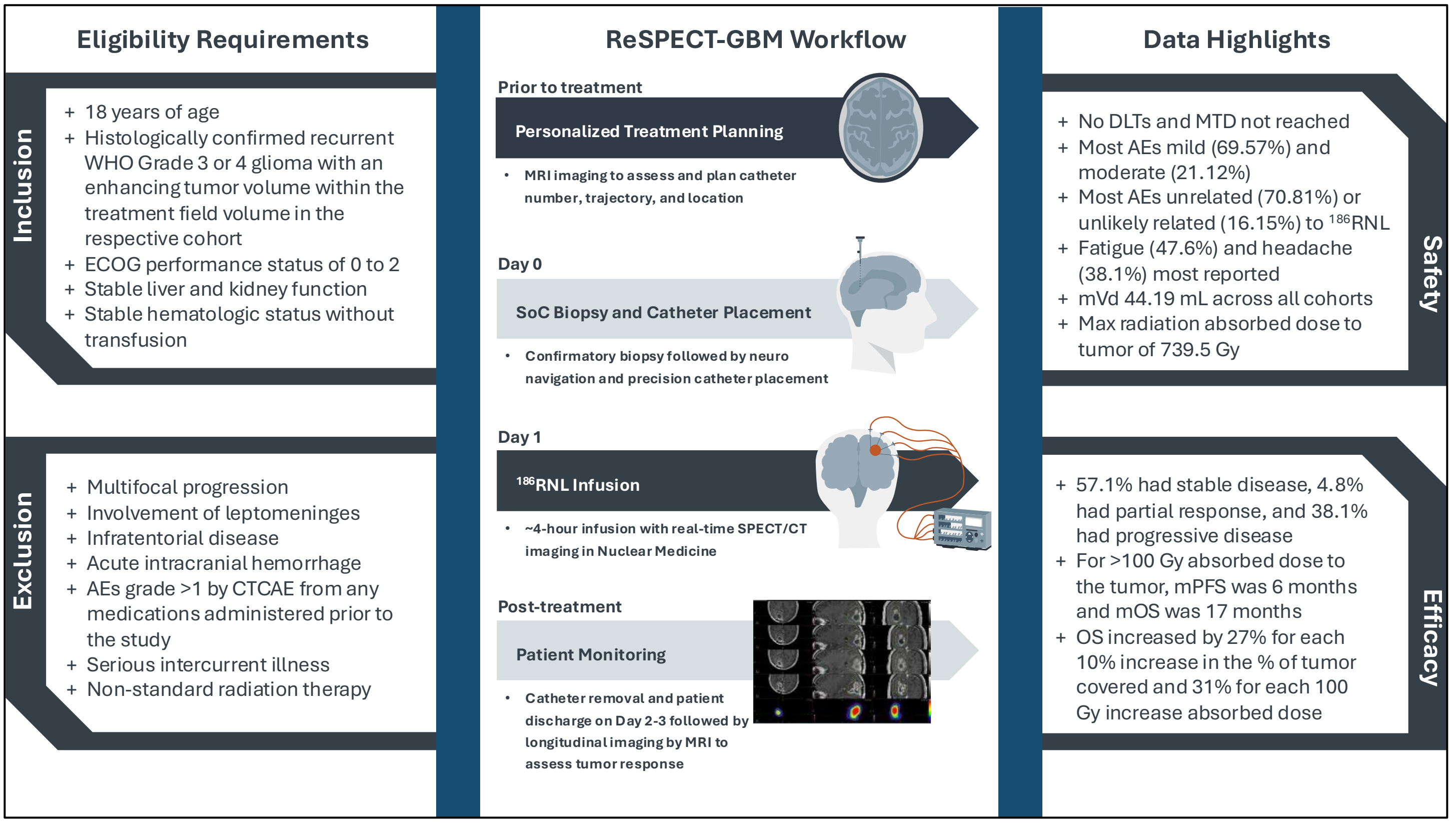


**Supplementary Figure 4. Infographic of study.**

| | Frequency (%) | | | | | |
|---|---|---|---|---|---|---|
| ***Outcome*** | ***Cohort 1*** | ***Cohort 2*** | ***Cohort 3*** | ***Cohort 4*** | ***Cohort 5*** | ***Cohort 6*** |
| 1-Resolved | 25 (100) | 34 (100) | 26 (100) | 20 (100) | 17 (100) | 33 (89.2) |
| 2-Resolved with Tx | 0 (0) | 0 (0) | 0 (0) | 0 (0) | 0 (0) | 4 (10.8) |
| Total | 25 | 34 | 26 | 20 | 17 | 37 |

**Supplementary Table 1. Adverse event outcome by cohort.**

| | Frequency (%) | | | | | | |
|---|---|---|---|---|---|---|---|
| ***SAE*** | ***Cohort 1*** | ***Cohort 2*** | ***Cohort 3*** | ***Cohort 4*** | ***Cohort 5*** | ***Cohort 6*** | ***Total*** |
| **Yes** | 0 (0) | 2 (5.9) | 0 (0) | 4 (20) | 1 (5.9) | 3 (8.1) | 10 |
| **No** | 25 (100) | 32 (94.1) | 26 (100) | 16 (80) | 16 (94.1) | 34 (91.9) | 149 |

**Supplementary Table 2. Severe adverse event by cohort.**

| ***Adverse Event*** | ***Cohort 1*** | ***Cohort 2*** | ***Cohort 3*** | ***Cohort 4*** | ***Cohort 5*** | ***Cohort 6*** | ***Frequency (%)*** |
|---|---|---|---|---|---|---|---|
| Anorexia | 0 | 0 | 0 | 1 | 0 | 0 | 1 (4.8) |
| Avascular necrosis | 0 | 0 | 0 | 0 | 0 | 1 | 1 (4.8) |
| Edema cerebral | 0 | 1 | 0 | 0 | 0 | 1 | 2 (9.5) |
| Hypersomnia | 0 | 0 | 0 | 1 | 0 | 0 | 1 (4.8) |
| Localized edema | 0 | 0 | 0 | 1 | 0 | 0 | 1 (4.8) |
| Lung infection | 0 | 0 | 0 | 0 | 0 | 1 | 1 (4.8) |
| Seizure | 0 | 1 | 0 | 1 | 1 | 0 | 3 (14.3) |

**Supplementary Table 3. All serious adverse events by cohort.**

| ***Model*** | ***Variable[1]*** | ***Coefficient±SE*** | ***Fold Change*** | ***95% CI*** | ***p-value*** |
|---|---|---|---|---|---|
| **1** | Age | 0.003±0.017 | 1.003 | 0.97, 1.037 | 0.85 |
| | Baseline ECOG Performance Status | -0.1±0.259 | 0.905 | 0.545, 1.504 | 0.7 |
| | Volume Administered | -0.003±0.048 | 0.997 | 0.908, 1.094 | 0.94 |
| | Baseline Tumor Volume | 0.004±0.012 | 1.004 | 0.98, 1.029 | 0.73 |
| | Ratio of Treated to Total Tumor Volume (%)/10 | 0.146±0.049 | 1.157 | 1.052, 1.274 | 0.003 |
| **2** | Age | 0.012±0.019 | 1.012 | 0.975, 1.05 | 0.53 |
| | Baseline ECOG Performance Status | -0.138±0.273 | 0.871 | 0.51, 1.488 | 0.61 |
| | Volume Administered | -0.023±0.054 | 0.978 | 0.88, 1.087 | 0.68 |
| | Baseline Tumor Volume | 0.01±0.014 | 1.01 | 0.982, 1.039 | 0.49 |
| | Total Dose (Gy) in Distribution volume/100 | 0.2±0.078 | 1.222 | 1.048, 1.424 | 0.01 |

**Supplementary Table 4. Progression free survival Accelerated Failure Time model.**

| ***Model*** | ***Variable*** | ***Coefficient±SE*** | ***Fold Change*** | ***95% CI*** | ***p-value*** |
|---|---|---|---|---|---|
| **1** | Age | -0.068±0.01 | 0.934 | 0.916, 0.953 | <0.001 |
| | Baseline ECOG Performance Status | 0.443±0.158 | 1.558 | 1.143, 2.123 | 0.005 |
| | Volume Administered | 0.014±0.032 | 1.015 | 0.952, 1.081 | 0.66 |
| | Baseline Tumor Volume | -0.004±0.009 | 0.996 | 0.979, 1.013 | 0.61 |
| | Ratio of Treated to Total Tumor Volume (%)/10 | 0.256±0.033 | 1.292 | 1.211, 1.377 | <0.001 |
| **2** | Age | -0.082±0.018 | 0.921 | 0.89, 0.954 | <0.001 |
| | Baseline ECOG Performance Status | 0.557±0.293 | 1.746 | 0.982, 3.102 | 0.06 |
| | Volume Administered | 0.064±0.054 | 1.066 | 0.96, 1.185 | 0.23 |
| | Baseline Tumor Volume | -0.004±0.015 | 0.996 | 0.966, 1.026 | 0.79 |
| | Total Dose (Gy) in Distribution volume/100 | 0.255±0.085 | 1.291 | 1.092, 1.526 | 0.003 |

**Supplementary Table 5. Overall survival Accelerated Failure Time model.**

| ***Cohort*** | ***Step*** | ***Rate per catheter [ul/min (mL/hour)]*** | ***Minimum Interval (minutes)*** |
|---|---|---|---|
| **1, 2, 3** | 1 | 1 (0.06) | 20 |
| | 2 | 2 (0.12) | 20 |
| | 3 | 5 (0.30) | 20 (additional as required) |
| **4** | 1 | 1 (0.06) | 20 |
| | 2 | 2 (0.12) | 20 |
| | 3 | 5 (0.30) | 20 |
| | 4 | 10 (0.60) | 20 (additional as required) |
| **5, 6** | 1 | 1 (0.06) | 20 |
| | 2 | 2 (0.12) | 20 |
| | 3 | 5 (0.30) | 20 |
| | 4 | 10 (0.60) | 20 |
| | 5 | 15 (1.20) | 20 (additional as required) |
| **6** | 1 | 5 (0.30) | 10 |
| | 3 | 10 (0.60) | 10 |
| | 4 | 15 (1.20) | 10 |
| | 5 | 20 (1.20) | 10 (additional as required) |

**Supplementary Table 6. Infusion rate per cohort.** Infusion rates of $^{186}$RNL started at 1 ul/min in lower Cohorts to confirm safety and tolerability and increased stepwise, once safety profiles were confirmed.

| Cohort | Subject Number | Catheters per Treatment Plan | Failed Catheter(s) | Total Infusion Time |
|---|---|---|---|---|
| 1 | 01-001 | 1 | 0 | 3.0 |
| 1 | 01-002 | 1 | 0 | 2.9 |
| 1 | 01-003 | 1 | 0 | 2.9 |
| 2 | 01-004 | 1 | 0 | 5.0 |
| 2 | 01-005 | 1 | 0 | 5.8 |
| 2 | 01-007 | 1 | 0 | 5.2 |
| 3 | 01-008 | 1 | 0 | 0.3 |
| 3 | 01-010 | 1 | 0 | 9.0 |
| 3 | 01-011 | 1 | 0 | 9.3 |
| 4 | 01-012 | 1 | 0 | 2.2 |
| 4 | 01-013 | 2 | 0 | 5.5 |
| 4 | 01-014 | 2 | 0 | 5.5 |
| 5 | 01-016 | 2 | 0 | 0.1 |
| 5 | 01-017 | 3 | 0 | 3.5 |
| 5 | 01-018 | 3 | 0 | 2.1 |
| 6 | 01-019 | 2 | 1 | 8.4 |
| 6 | 01-020 | 3 | 0 | 3.2 |
| 6 | 01-021 | 4 | 1 | 2.9 |
| 6 | 02-001 | 4 | 0 | 3.8 |
| 6 | 02-002 | 2 | 0 | 4.5 |
| 6 | 02-003 | 2 | 1 | 8.1 |

**Supplementary Table 7. Catheters used/failed and infusion time per cohort/patient.**

| Cohort | Subject | Average absorbed dose to tumor (Gy) |
|---|---|---|
| 1 | 01-001 | 143.00 |
| 1 | 01-002 | 396.30 |
| 1 | 01-003 | 56.40 |
| 2 | 01-004 | 8.90 |
| 2 | 01-005 | 334.00 |
| 2 | 01-007 | 21.85 |
| 3 | 01-008 | 69.50 |
| 3 | 01-010 | 38.17 |
| 3 | 01-011 | 592.70 |
| 4 | 01-012 | 57.20 |
| 4 | 01-013 | 36.00 |
| 4 | 01-014 | 418.60 |
| 5 | 01-016 | 516.90 |
| 5 | 01-017 | 336.60 |
| 5 | 01-018 | 414.20 |
| 6 | 01-019 | 93.20 |
| 6 | 01-020 | 424.60 |
| 6 | 01-021 | 62.60 |
| 6 | 02-001 | 342.70 |
| 6 | 02-002 | 739.50 |
| 6 | 02-003 | 584.20 |

**Supplementary Table 8. Average absorbed dose per cohort/patient.**

# A Dual Phase 1/2, Investigator Initiated Study to Determine the Maximum Tolerated Dose, Safety, and Efficacy of $^{186}$Rhenium Nanoliposomes ($^{186}$RNL) in Recurrent Glioma

**Principal Investigator:** Andrew Brenner, MD, PhD
**Lead Site** Institute for Drug Development
UT Health San Antonio
Mays Cancer Center
7979 Wurzbach Road, 4th floor
San Antonio, TX 78229
Tel. (210) 450-5936
Cell (210) 667-5860
Fax. (210) 692-7502
E-mail: brennera@uthscsa.edu

John Floyd, MD
University Health System
4502 Medical Drive
San Antonio, TX 78229
Tel. (210) 567-5625
E-mail: floyd@uthscsa.edu

## Participating Sites Principal Investigators:

**Toral Patel, MD**
UT Southwestern Medical Center
5323 Harry Hines Blvd.
Dallas, TX 75390
Tel. (214) 648-6403
E-mail: toral.patel@utsouthwestern.edu

**Jeffrey Weinberg, MD, FAANS, FACS**
UT MD Anderson Cancer Center
1400 Holcombe Blvd.
Department of Neurosurgery, Unit 442
Houston, TX 77030
Tel. (713) 792-2400
E-mail: jweinberg@mdanderson.org

**Sub-Investigators:** See FDA 1572

**Sponsor:**

Plus Therapeutics, Inc.
12500 Network Blvd., Suite 207
San Antonio, TX 78249
Tel. (210) 974-6900

**$^{186}$RNL Manufacturing:**

**RadioMedix, Inc.**
9701 Richmond Ave. #222
Houston, TX 77042
**Tel. (713) 358-6500**

## Contacts:

PLUS THERAPEUTICS MAIN CONTACT:
Norman LaFrance, MD, ME, FACP, FACNP, FACNM
Chief Medical Officer, SVP
(215) 808-0955
nlafrance@plustherapeutics.com

PLUS THERAPEUTICS MEDICAL MONITOR/SAFETY REPORTING (SAE) CONTACT:
Norman LaFrance, MD, ME, FACP, FACNP, FACNM
Chief Medical Officer, SVP
Tel. (215) 808-0955
safety_inbox@plustherapeutics.com

PLUS THERAPEUTICS CLINICAL OPERATIONS CONTACT
BACKUP SAFETY AND MAIN CONTACT:
Melissa Moore, PhD
Senior Director, Clinical Research
Tel. (347) 570-3338
mmoore@plustherapeutics.com

PLUS THERAPEUTICS QUALITY ASSURANCE AND REGULATORY CONTACT:
Erika Butler, MS
Senior Manager, Quality Assurance
(210) 974-6919
ebutler@plustherapeutics.com

## IND# 116117

## Protocol Amendment Version 20.0 dated: 29Sep22

# Table of Contents


1. INTRODUCTION ..... 13
  1.1. Scientific Background ..... 13
    1.1.1. Glioma ..... 13
    1.1.2. Conventional Radiation Treatment ..... 14
    1.1.3. Brachytherapy and Targeted Radiotherapy ..... 14
    1.1.4. Convection Enhanced Delivery (CED) ..... 15
    1.1.5. Rhenium-186 Radionuclide ..... 16
    1.1.6. Rhenium-labeled Nanoliposomes ..... 16
  1.2. Preclinical Data ..... 17
    1.2.1. Establishing Studies ..... 17
    1.2.2. GLP Safety Assessment ..... 18
  1.3. Clinical Data ..... 19
    1.3.1. Phase 1 First-in-Human Study in Adults with Recurrent Glioma ..... 19
    1.3.2. Dose Escalation ..... 20
    1.3.3. Primary Endpoints ..... 20
    1.3.4. Secondary Endpoints ..... 21
    1.3.5. Dosimetry ..... 21
    1.3.6. Retreatment ..... 21
  1.4. Other Clinical Studies ..... 22
2. RATIONALE FOR THE STUDY ..... 22
  2.1. Rationale for selection of the subject population ..... 23
  2.2. Study Compliance ..... 23
3. OBJECTIVES OF THE STUDY ..... 23
  3.1. Primary Objectives ..... 23
    3.1.1. Phase 1 ..... 23
    3.1.2. Phase 2 ..... 23
  3.2. Secondary Objectives ..... 23
    3.2.1. Phase 1 ..... 23
    3.2.2. Phase 2 ..... 23
4. STUDY DESIGN AND METHODS ..... 24
  4.1. Phase 1 ..... 24
    4.1.1. Overview ..... 24

4.1.2. Subject Assignment Methods ....................26
4.1.3. Statistical Analysis ....................27
4.2. Phase 2 ....................27
4.2.1. Overview ....................27
4.2.2. Clinical Endpoints ....................27
4.2.3. Therapy ....................27
4.2.4. Disease Assessment ....................28
4.2.5. Imaging Parameters ....................28
4.2.6. Statistical Analysis ....................28
5. PATIENT POPULATION ....................29
5.1. Inclusion Criteria ....................29
5.2. Exclusion Criteria ....................30
6. PROCEDURES ....................31
6.1. Summary ....................31
6.2. Screening ....................31
6.2.1. Informed Consent ....................32
6.2.2. Procedures ....................32
6.3. Treatment ....................33
6.3.1. Evaluations ....................33
6.3.2. Procedures ....................33
6.4. End of Study, Early Study Termination, and Survival Follow-up ....................38
6.4.1. End of Study or Early Study Termination ....................38
6.4.2. Survival Follow-up ....................39
6.5. Safety Procedures ....................39
6.5.1. Physical Examination ....................39
6.5.2. Vital Signs ....................39
6.5.3. Disease Assessment ....................39
7. MANAGEMENT OF INTERCURRENT EVENTS ....................40
7.1. Grading of Toxicity ....................40
7.2. Monitoring and Treatment of Toxicity ....................40
7.3. Adverse Events ....................40
7.3.1. Defining Adverse Events ....................40
7.3.2. Documenting All Adverse Events ....................42
7.3.3. Reporting and Documenting Serious Adverse Events ....................43

7.3.4. Follow Up of Adverse Events ........ 44
7.4. Concomitant and excluded therapy ........ 44
8. REMOVING SUBJECTS FROM THE STUDY ........ 44
8.1. Criteria for termination ........ 44
8.2. Documentation ........ 45
8.3. Procedures for subjects who withdraw early ........ 45
8.4. Replacement of subjects ........ 45
9. CONDITIONS FOR INITIATING, MODIFYING, OR TERMINATING THE STUDY ........ 45
9.1. Institution Review ........ 45
9.2. Informed Consent ........ 46
9.3. Modifications ........ 46
9.4. Deviations ........ 46
9.5. Termination ........ 46
10. INVESTIGATOR'S RESPONSIBILITIES ........ 46
10.1. Responsibilities/Performance ........ 46
10.2. Confidentiality ........ 47
10.3. Institutional Review ........ 47
10.4. Informed Consent and Permission to Use Protected Health Information ........ 47
10.5. Source Documentation and Investigator Files ........ 47
10.5.1. Subject Exclusion Log ........ 48
10.6. Data Safety Monitoring ........ 48
11. REFERENCES ........ 50

## List of Tables

Table 1. Phase 1 Dose Escalation plan for $^{186}$RNL. Cohort 6b utilized the same volume and dose as Cohort 6a but with an increase in maximum flow rate to 20 microliters/minute. ........ 20

Table 2: Modified Fibonacci dose escalation scheme. Cohort 6b utilized the same volume and dose as Cohort 6a but with an increase maximum flow rate to 20 microliters/min. ........ 26

# List of Appendices

APPENDIX A, SCHEDULE OF EVENTS ........ 533
APPENDIX B: EASTERN COOPERATIVE ONCOLOGY GROUP (ECOG) PERFORMANCE SCORE SCALE ........ 544
APPENDIX C: RANO TUMOR RESPONSE ........ 555
APPENDIX D: PATIENT SAFETY SHEET ........ 566
APPENDIX E: CATHETER AND INFUSION RATE DATA ........ 587

PROTOCOL SIGNATURE PAGE

I have read and understand the contents of this clinical protocol for "A Dual Phase 1/2, Investigator Initiated Study to Determine the Maximum Tolerated Dose, Safety, and Efficacy of $^{186}$Rhenium Nanoliposomes ($^{186}$RNL) in Recurrent Glioma" and will adhere to the study requirements as presented, including all statements regarding confidentially. In addition, I will conduct the study in accordance with current international conference on harmonization (ICH) guidance, Good Clinical Practice (GCP) guidance, the Declaration of Helsinki, US Food and Drug Administration (FDA) regulations and local IRB and legal requirements.

**Agreed to by (Investigator):**

____________________________________________

**Printed Name - Investigator**

____________________________________________

**Institution**

____________________________________________

**Signature – Investigator**

__________________________________

**Date**

**Agreed to by (Sponsor):**

____________________________________________

**Printed Name – Sponsor**

____________________________________________

**Signature – Sponsor**

__________________________________

**Date**

**SYNOPSIS**

| | |
|---|---|
| **Study Title:** | A Dual Phase 1/2, Investigator Initiated Study to Determine the Maximum Tolerated Dose, Safety, and Efficacy of $^{186}$Rhenium Nanoliposomes ($^{186}$RNL) in Recurrent Glioma |
| **Indication:** | Recurrent glioma progressing after conventional treatment. |
| **Primary Objectives:** | Phase 1:<br>To determine the maximum tolerated dose of $^{186}$RNL by convection enhanced delivery (CED) at the time of planned stereotactic biopsy, when necessary, as standard of care.<br><br>Phase 2:<br>To assess overall survival (OS) following $^{186}$RNL administration by convection enhanced delivery (CED) in patients with recurrent glioma. |
| **Secondary Objectives:** | Phase 1:<br><br>To assess the safety of single dose $^{186}$RNL by CED.<br>To assess the dose distribution of $^{186}$RNL by CED.<br>To determine the overall response rate by Radiographic Assessment in Neuro-Oncology (RANO) criteria following $^{186}$RNL treatment.<br>To determine disease specific progression-free survival after $^{186}$RNL treatment.<br><br>Phase 2:<br>To assess the safety and tolerability of $^{186}$RNL by the National Cancer Institute (NCI) Common Terminology Criteria for Adverse Events (CTCAE) v4.0 criteria. Safety and tolerability will be defined by the percent of participants experiencing ≥ Grade 3 AE/SAE.<br>To determine the objective response rate (ORR) from the date of complete or partial response or Serious Treatment-Emergent Adverse Events (Safety and Tolerability) *up to 3 years*.<br>To determine progression free survival at 6 months (PFS-6) as measured from the initiation of study treatment until the date of first documented progression by modified RANO criteria (recognizing the potential of pseudo progression significantly complicating the use of the RANO criteria) or date of death from any cause *up to 6 months*.<br>To determine progression free survival (PFS) as measured from the initiation of study treatment until the date of first documented progression by modified RANO criteria (recognizing the potential of pseudo progression significantly complicating the use of the RANO criteria) where progression is defined as >25% in the sum of products of the perpendicular diameters of CE lesions, evidence of new lesion(s), or date of death from any cause, whichever comes first, *up to 3 years*.<br>Evaluate assessing quality of life using a Quality-of-Life Questionnaire (developed in collaboration with FDA, Investigators and established glioblastoma patient advocacy groups). |

| | |
|---|---|
| **Study Design:** | <u>Phase 1:</u><br>Single arm, prospective study utilizing a modified Fibonacci dose escalation scheme.<br><br><u>Phase 2:</u><br>Single arm, prospective study utilizing a non-DLT dose obtained from the dose escalation portion of IND 116117, NIH-NCI Grant (22.3 mCi (total $^{186}$RNL activity) at a concentration of 2.5 mCi/mL and 8.8 mL total volume). |
| **Duration:** | Subjects will remain on study until disease progression by RANO criteria (recognizing the potential of pseudo progression significantly complicating the use of the RANO criteria) or PI decision in the best interest of the patient. |
| **Planned Total Sample Size:** | 55 subjects. An estimated 21 subjects will be enrolled for the dose escalation, and determination of MTD/RP2D. An additional 34 evaluable patients at the recommended Phase 2 dose (22.3 mCi (total $^{186}$RNL activity) at a concentration of 2.5 mCi/mL and 8.8 mL total volume). |

| | |
|---|---|
| **Inclusion Criteria:** | 1. At least 18 years of age.<br>2. Ability to understand the purposes and risks of the study and has signed a written informed consent form approved by the investigator's IRB/Ethics Committee.<br>3. Histologically confirmed Grade III/IV recurrent Glioma (following 2021 WHO CNS5 glioma nomenclature, e.g., Astrocytoma, IDH-mutant grade 3 or 4; Glioblastoma, IDH-wildtype grade 4).<br>4. Progression by RANO criteria or other clinically accepted neurooncology evaluation, following standard treatment options with known survival benefit for any recurrence (e.g., surgery, temozolomide, radiation, and tumor treating fields). Patient may be included in study if medically unable or unwilling to follow standard treatment options for any recurrence.<br>5. Patients who receive treatment with antiepileptic medications must have a two-week history of stable dose of antiepileptic without seizures prior to study start (dosing).<br>6. Patients with corticosteroid requirements to control cerebral edema must be maintained at a stable or decreasing dose for a minimum of two weeks without progression of clinical symptoms (Section 6.3.2.7) prior to study start (dosing).<br>7. Patients with Grade III/IV Glioma (following 2021 WHO CNS5 glioma nomenclature, e.g., Astrocytoma, IDH-mutant grade 3 or 4; Glioblastoma, IDH-wildtype grade 4) which falls within the treatment field volume.<br>8. ECOG performance status of 0 to 2; Karnofsky Performance Status ≥ 60.<br>9. Life expectancy of at least 2 months.<br>10. Acceptable liver function:<br>a. Bilirubin ≤ 1.5 times upper limit of normal<br>b. AST (SGOT) and ALT (SGPT) ≤ 3.0 times upper limit of normal (ULN)<br>11. Acceptable renal function:<br>a. Serum creatinine ≤1.5xULN<br>12. Acceptable hematologic status (without hematologic support):<br>a. ANC ≥1000 cells/uL<br>b. Platelet count ≥100,000/uL<br>c. Hemoglobin ≥9.0 g/dL<br>13. All women of childbearing potential must have a negative serum pregnancy test and male and female subjects must agree to use effective means of contraception (for example, surgical sterilization or the use of barrier contraception with either a condom or diaphragm in conjunction with spermicidal gel or an IUD) with their partner from entry into the study through 6 months after the last dose. |
| **Exclusion Criteria:** | 1. The subject has evidence of acute intracranial or intratumoral hemorrhage either by magnetic resonance imaging (MRI) or computerized tomography (CT) scan. Subjects with resolving hemorrhage changes, punctate hemorrhage, or hemosiderin are eligible.<br>2. The subject is unable or contraindicated to undergo MRI scan (e.g., has pacemaker or medically unstable).<br>3. The subject has not recovered to CTCAE v4.0 Grade ≤1 from AEs (except alopecia, anemia, and lymphopenia) due to antineoplastic agents, investigational drugs, or other medications that were administered prior to study.<br>4. The subject is pregnant or breast-feeding. |

<table>
<tr>
<td>Exclusion Criteria cont.:</td>
<td>
5. The subject has serious intercurrent illness, as determined by the treating physician, which would compromise either patient safety or study outcomes such as:<br>
• hypertension (two or more blood pressure readings performed at screening of >150 mmHg systolic or >100 mmHg diastolic) despite optimal treatment<br>
• active medically significant infection unresponsive to antibiotics (e.g., non-healing wound, ulcer), uncontrolled systemic infection, or bone fracture<br>
• clinically significant cardiac arrhythmias not controlled by appropriate medications<br>
• untreated hypothyroidism<br>
• symptomatic congestive heart failure or unstable angina pectoris within 3 months prior to study drug<br>
• myocardial infarction, stroke, or transient ischemic attack within 6 months prior to study drug<br>
• known active malignancy (other than glioma) except non-melanoma skin cancer or carcinoma in-situ in the cervix unless PI determines it would not impact patient safety or efficacy determinations<br>
6. The subject has inherited bleeding diathesis or coagulopathy with the risk of bleeding.<br>
7. The subject has received any of the following prior anticancer therapy:<br>
• Prior treatment with Bevacizumab<br>
• Non-standard radiation therapy such as brachytherapy, systemic radioisotope therapy, or intra-operative radiotherapy (IORT) to the target site<br>
• Radiation therapy within 12 weeks of screening<br>
• Systemic therapy (including investigational agents and small-molecule kinase inhibitors) or non-cytotoxic hormonal therapy (e.g., tamoxifen) within 14 days or 5 half-lives, whichever is shorter, prior to study start (dosing)<br>
• Biologic agents (antibodies, immune modulators, vaccines, cytokines) within 21 days prior to study start (dosing)<br>
• Nitrosoureas or mitomycin C within 42 days, or metronomic/protracted low-dose chemotherapy within 14 days, or other cytotoxic chemotherapy within 28 days, prior to study start (dosing)<br>
• Prior treatment with carmustine wafers<br>
• Patients who are currently receiving any other investigational agents and/or who have received an investigational agent in 28 days prior to study start (dosing)<br>
8. Multifocal progression or involvement of the leptomeninges.<br>
9. Psychiatric illness/social situations that would limit compliance with the study requirements<br>
10. Infratentorial disease<br>
11. The subject has a tumor located within 1-2 cm of a ventricle AND it is determined by the surgeon, PI, and sponsor to be a risk for drug extravasation to the subarachnoid space if given catheter placement and drug administration.<br>
12. Phase 2 only: The subject should have a tumor volume of $\leq 20$ cm$^3$ to be included in the Phase 2 portion of the study. Subjects with tumor volumes of greater than 20 cm$^3$ are excluded from the Phase 2 portion of the study.
</td>
</tr>
</table>

| | |
|---|---|
| **Safety:** | Safety endpoints:<br>• Incidence and severity of adverse events<br>• Changes in lab parameters, vital signs, and weight |
| **Procedures (Summary):** | One to four catheters will be placed within the tumor using stereotactic guidance. Once the patient has adequately recovered from the procedure as determined by the neurosurgeon, $^{186}$RNL will be infused through the catheters at the predetermined dose. Whole body anterior and posterior planar and SPECT brain scintigraphic imaging will then be obtained at predefined time points to visualize the distribution of the $^{186}$RNL as well as calculate the actual dose retained within the tumor. Patients will be monitored longitudinally for evidence of toxicity and response by MRI. |

# 1. INTRODUCTION

## 1.1. Scientific Background

### 1.1.1. Glioma

In 2022, it is estimated there will be approximately 25,050 new brain and other central nervous system cases diagnosed (1.3% of all cancers) and 18,280 deaths (3.0% of all cancer related deaths) (NCI 2022). The 2021 World Health Organization Classification of CNS Tumors (WHO CNS5) classifies Gliomas, Glioneuronal Tumors, and Neuronal Tumors into 6 different families: (1) Adult-type diffuse gliomas, (2) Pediatric-type diffuse low-grade gliomas, (3) Pediatric-type diffuse high-grade gliomas, (4) Circumscribed astrocytic gliomas, (5) Glioneuronal and neuronal tumors; and (6) Ependymomas (Louis et al. 2021). Adult-type diffuse gliomas include Astrocytoma, IDH-mutant; Oligodendroglioma, IDH-mutant, and 1p/19q-codeleted; and Glioblastoma, IDH-wildtype. Astrocytoma, IDH-mutant is graded 2-4. Glioblastoma, IDH-wildtype is graded 4, makes up most primary brain tumors diagnosed in the adult neurooncology setting, and is the most aggressive with dismal outcomes (Ostrom et al. 2020; Louis et al. 2021).

For GBM (used here to indicate pre-WHO CNS5 changes, and therefore include both grade 4 astrocytoma, IDH-mutant and grade 4 glioblastoma, IDH-wildtype), the historical, general 5-year survival rate is 5.5% (Ostrom et al. 2020). Durable high-grade glioma therapies remain elusive, and the surgery, radiation, and temozolomide standard-of-care therapies for newly diagnosed glioblastoma remain largely unchanged (Stupp et al. 2005; Stupp et al. 2015). The poor survival is attributable partly to the nature of the tumor – the infiltrative nature of GBM results in difficulty eliminating microscopic disease despite macroscopic gross-total resection, with >90% of patients having recurrence at the original tumor location (Hou et al. 2006). Furthermore, the location of the tumor makes drug delivery difficult, with only small or lipophilic molecules able to cross the blood brain barrier to reach the tumor. Of those agents that can reach the tumor, GBM has shown to be resistant to most cytotoxic agents and to quickly develop resistance when initially sensitive.

#### 1.1.1.1. Standard Treatment for High Grade Glioma

Currently, front-line treatment consists of a multi-modality approach that includes maximal surgical resection, adjuvant radiation therapy of 54-60 Gy with concurrent temozolomide at 75 $mg/m^2$, and 6 months of single agent temozolomide up to 200 $mg/m^2$ with tumor treatment fields (TTF) (Stupp et al. 2005; Stupp et al. 2015). Temozolomide (a prodrug) is a rapidly and non-enzymatically converted to the active alkylating metabolite MTIC ((methyl-triazene-1-yl)-imidazole-4-carboxamide). The cytotoxic effects of MTIC are manifested through alkylation of DNA at the O6, N7 guanine positions.

Radiation remains the most effective component of the combined approach, with multiple randomized studies showing a 5-month improvement in survival with XRT alone, compared to an additional 2.5 months with the addition of chemotherapy and 3 months for TTF (Chang et al. 2007). Once a patient fails standard front-line therapy, prognosis is extremely poor. The only currently approved therapeutic for salvage treatment is bevacizumab, a recombinant humanized monoclonal antibody against VEGF with an associated median overall survival (OS) time of ~7.4-

9.2 months (Friedman et al. 2009; Cloughesy et al. 2020). After bevacizumab failure, survival is only approximately 120 days (Quant et al. 2009).

Thus, as a disease with few treatment options, poor prognosis, and a pattern of recurrent local progression, novel, effective treatments for gliomas remain a medically unmet need and an appropriate indication for the study of $^{186}$RNL.

### 1.1.2. Conventional Radiation Treatment

Radiation therapy remains an essential component of treatment for most malignancies, including primary brain tumors. Theoretically, any tumor can be controlled if a sufficient dose of radiation is delivered to the tumor. The main limiting factor in delivering a tumoricidal dose is the toxicity to surrounding normal tissue. As the traditional x-ray radiation beam passes through the skull and brain to reach the tumor, it is absorbed by the body and shows exponential decrease in the dose delivered with tissue depth.

To limit the exposure of the surrounding normal brain and maximize exposure of the tumor, techniques such as Tomotherapy are used. Tomotherapy, or Helical Tomotherapy, is a form of computed tomography (CT) guided Intensity Modulated Radiation Therapy (IMRT). The Tomotherapy treatment system delivers radiation therapy with a spiral delivery pattern. Photon radiation is produced by a linear accelerator, which travels around the patient and moves in unison with a device called a multi-leaf collimator, or MLC, which shapes the beam. At the same time, the patient bed is also moving to guide the patient slowly through the center of the ring. Even accounting for this more conformal application, doses are limited to less than 80 Gy (Werner-Wasik et al. 1996), and often less than 50 Gy.

### 1.1.3. Brachytherapy and Targeted Radiotherapy

Brachytherapy is an alternate form of radiotherapy where a radiation source is placed inside or next to the area requiring treatment. Biologically targeted radiotherapy, as defined here, include therapies that bind using delivery vehicles that either bind specifically to endogenous targets (Sgouros et al. 2020). A radiation source suitable for brachytherapy and biologically targeted radiotherapy should have an intermediate diffusion range in tissues to concentrate the energy release inside the tumor while minimizing the damage to surrounding healthy tissues. Attention has therefore been focused to a large degree on beta emitting isotopes which have a tissue penetration depth of up to 11 mm. The most successful example has been in the use by systemic administration of radioactive iodine to treat thyroid cancer in addition to brachytherapy use of $^{131}$I. Radioactive $^{131}$I remains a powerful therapy for treatment of thyroid cancer. Every decay of $^{131}$I results in the emission of beta particles with an average range in tissue of 1 mm and a maximum range of 3 mm. Its success in thyroid cancer therapy is due to the natural affinity of thyroid cancer cells for iodine and the short-range field of therapy of the beta particles, resulting in the complete ablation of thyroid cancer with doses of nearly 1000 Gy, with limited toxicity to surrounding normal tissue. Furthermore, $^{131}$I is rapidly cleared from the body by the kidneys with no uptake by bone. $^{131}$I is widely available and relatively inexpensive because it is produced in a medical nuclear reactor, and most thyroid cancer patients in the United States receive $^{131}$I therapy following surgery for remnant thyroid tissue ablation and/or the treatment of metastatic thyroid cancer.

Recently, a new therapeutic radiopharmaceutical, lutetium-177 ($^{177}$Lu)-Dotatate, has been shown to increase survival in gastroenteropancreatic neuroendocrine tumors (Strosberg et al. 2017), leading to FDA approval. Additional therapeutics featuring beta- and alpha-emitting radionuclides attached to small molecules, proteins, peptides, antibodies, nanoparticles, and other targeting structures are in clinical trials (Sgouros et al. 2020).

Both examples represent well established avidity for the malignant target receptors. Although target avidity is a crucial to the efficacy of these radiopharmaceuticals, the challenge with these *systemically* administered radiopharmaceuticals is that because of the systemic route of administration, exposure to normal tissues and organs is unavoidable and the pharmacokinetics dictates the toxicity severity and normal organ and tissue dosimetry (absorbed radiation dose). Especially in cancers of the CNS, an effective means of precisely delivering these therapeutic radionuclides is lacking and, while systemically administered drug delivery, whether pharmaceutical or radiopharmaceuticals are significantly impacted and prevented by the CNS blood brain barrier (BBB). One way to manage and eliminate this challenge is to have a more efficient, direct locoregional delivery of the therapeutic radiopharmaceutical.

### 1.1.4. Convection Enhanced Delivery (CED)

One of the most effective methods of locally delivering therapeutic agents to the brain has been Convection Enhanced Delivery (CED) first introduced nearly 30 years ago (Bobo et al. 1994). As noted above, the blood brain barrier (BBB) represents a substantial challenge to developing effective treatments for tumors of the CNS. CED relies on bulk flow, or a hydrostatic pressure gradient, to distribute infusate through the interstitial spaces of the brain tissue. Based on the properties of bulk flow distribution, there are several defined and unique features of convective delivery to the nervous system. Because infusate is delivered directly into the brain parenchyma via a catheter, the BBB is bypassed, and specific regions can be targeted for therapeutic drug delivery and treatment (I. Mehta et al. 2012).

CED does not rely on diffusion, so small or large molecular weight substances can be distributed in a homogeneous manner over clinically relevant volumes. To date, almost all CED research has been directed at delivering chemotherapeutic agents to the brain (Vogelbaum and Iannotti 2012). Technological breakthroughs involving non-invasive imaging technology to guide placement, as well as effective methods of locally delivering therapeutic agents have come together to make this type of drug delivery a feasible approach.

Specific catheters have been developed for CED, are FDA cleared (April 2011), and commercially available (Appendix E). For example, the SmartFlow cannula (BrainLab) currently used in this protocol is an MRI-compatible injection catheter for use in the brain. The SmartFlow cannula is compatible with Brainlab's Neuronavigation System and VarioGuide Stereotactic Arm. Using the SmartFlow catheter with the VarioGuide system, neurosurgeons can navigate the SmartFlow catheter to the tumor under intra-procedural MRI guidance.

### 1.1.5. Rhenium-186 Radionuclide

Rhenium-186 ($^{186}$Re, half-life ~90 hours) is a reactor produced isotope with immense potential for medical therapy. It is in the same chemical family as Technetium-99m ($^{99m}$Tc), a radioactive tracer that is the most used isotope for diagnostic scintigraphic imaging in nuclear medicine. Like $^{99m}$Tc, $^{186}$Re is not taken up by bone and is readily cleared by the kidneys. While $^{186}$Re emits therapeutic beta particles, every 10th isotope decay also produces a 137 keV gamma photon optimal for quantitative imaging of its *in vivo* distribution for therapy evaluation. The emitted gamma photons have similar photon energy to those emitted by $^{99m}$Tc (140kev), allowing for imaging of the isotope within the body on standard nuclear imaging equipment available in routine medical practice.

The average $^{186}$Re beta particle path length in tissue of 1.8-2 mm is ideal for treatment of solid tumors and the half-life of ~90 hours is clinically meaningful. Therefore, the $^{186}$Re isotope has exciting potential in CED applications of local therapy of solid tumors. However, a carrier is needed to deliver the isotope to the brain and maintain its localization at the desired site, as otherwise it would quickly disperse and be carried away from the site of infusion by the circulatory system.

### 1.1.6. Rhenium-labeled Nanoliposomes

Liposomes are spherical, self-assembling vesicles made up of one (unilamellar) or more (multilamellar) naturally occurring lipid bilayers in a central compartment, which makes them ideal candidates as delivery vehicles for small molecules, proteins, nucleic acids, and imaging agents for therapeutic and diagnostic use (Liu et al. 2022). The bilayers are naturally occurring and nearly identical to the lipid membranes of normal cells, allowing for use of the natural degradation pathways in the human body.

Their properties make them well suited for drug delivery, as they can protect their encapsulated contents from degradation, extend the half-life of drugs, and actively or passively target their site, allowing for a decrease in systemic side effects despite increased drug doses (Liu et al. 2022). Furthermore, they are amenable to various administration routes, including parenteral, pulmonary, oral, transdermal, ophthalmic, and nasal (Liu et al. 2022). Several clinically approved products use liposomes as drug carriers, including Doxorubicin hydrochloride, Daunorubicin Amphotericin B, Cytarabine, Verteporfin, Morphine, MTP-PE, Bupivacaine, Vincristine Sulfate, Irinotecan hydrochloride trihydrate, Recombinant varicella-zoster virus glycoprotein E, and Amikacin sulfate (Liu et al. 2022). These treatments span a variety of indications, including cancer. Additionally, liposomes have been extensively used in the food and cosmetic industry.

Although larger liposomes can be manufactured, the most useful size range for drug carrier applications is 80-130 nm. Liposomes in the ~100 nm size range have been the most investigated carrier for CED drug delivery to the brain. These studies include the use of CED-delivery of nanoliposomes carrying chemotherapeutic agents directly to brain tumor, such as CPT-11 and topotecan.

To utilize nanoliposomes to carry radioisotopes, a method for the efficient loading of liposomes with the radioisotopes is required. Labelling of radiotherapeutic rhenium radionuclides to very

high levels of specific activity has been demonstrated (Phillips et al. 2012). This approach uses a proprietary encapsulation method using a custom lipophilic molecule, N, N-bis(2-mercaptoethyl)-N'N'-diethylethylene diamine (BMEDA), that chelates $^{186}$Re and carries it into the aqueous compartment of the liposome nanoparticles where it is irreversibly trapped using a well-established pH process. For treatment of locally invasive tumors, liposomal encapsulation of radiotherapeutics holds significant promise. While the liposomal encapsulation ensures the retention within the tissue, the emission of radiation beta particles allows therapeutic effect well beyond the individual cell. The ability to treat the whole tumor is greatly enhanced by the 2 mm average path length of the beta particle radiation which compensates for mild inhomogeneities in the CED dispersion of the nanoparticles within the tumor. The 2 mm pathlength means that therapy delivered to one cell has the potential of moving through 80 cell diameters, since an average cell diameter is 25 microns (25 x 80 cell diameters = 2 mm pathlength).

The final investigational product ($^{186}$RNL), a BMEDA-chelated-$^{186}$Rhenium encapsulated within liposomes, allows the $^{186}$Re to be directly delivered to the site of the tumor through CED and maintain localization at the site of infusion.

## 1.2. Preclinical Data

### 1.2.1. Establishing Studies

Preclinical models of glioma show $^{186}$RNL can eradicate transplanted tumor cells when > 100 Gy of radiation is delivered (Phillips et al. 2012). To characterize the potential utility of nanoliposomes in the 87U intracranial glioma xenograft animal model, as well as the ability to deliver an appropriate field of therapy, intra-tumoral infusions of technetium-99m-labeled liposomes were performed followed by SPECT/CT imaging. On planar image quantitative analysis, approximately 50-65% of the injected $^{99m}$Tc-activity was retained within the site of injection at 2, 4, and 20 h post injection using a 100 µL injection volume (Phillips et al. 2012).

Subsequently, characterization of the tolerability of progressive doses of $^{186}$RNL in the 87U GBM xenograft model was performed. An initial pilot experiment with doses administered up to 560 Gy (n = 6) showed 2 animals at the lower doses died at 19- and 22-days following treatment, while animals at the highest doses survived through sacrifice at day 37 post-treatment without weight loss, neurological deficit, or other evidence of toxicity. Histologic analysis by a neuropathologist showed no evidence of residual tumor.

Progressive extension of the dosing range was performed in two successive Cohorts with administration of 18 to 1,845 Gy (n = 12 in treatment group, 8 controls), following animals 120 days post-treatment before sacrifice. It was consistently observed within these Cohorts that animals tolerated all doses without evidence of toxicity and no maximum tolerable dose was reached.

Tumors were followed by both non-invasive bioluminescence luciferase imaging and MRI to determine if a dose dependent effect could be observed. A separation in bioluminescence between animals treated with less than or equal to 100 Gy and those at higher doses was seen within seven days of treatment. This became statistically significant by 11 days post treatment ($X^2$ (1, N=19) = 4.8, p = 0.029) at which time 11 treated rats were alive as well as all 8 controls. The separation in

bioluminescence continued thereafter. In addition, many of the rats had a loss of luciferase activity to background levels suggestive of a complete eradication of tumor and MRI evidence of response.

Control rats (n = 14) uniformly had a median survival time of 49 days in each of the 3 experiments (95% CI 44-53 days) from intracranial injection. On intent to treat analysis without regard to dose, animals treated with $^{186}$RNL had a median survival of 126 days (95% CI 78.4-173 days), with limitation in survival of treated animals only being an effect of sacrifice at the end of the experiment. Log-rank Analysis between these two groups was highly significant ($p = 0.0013$). When survival length by dose was analyzed, there was a recognizable separation beginning at a dose of 100 Gy. Survival analysis for treatment > 100 Gy as compared to less or none with log-rank analysis showed even greater significance ($p < 0.0001$).

Brains were collected from sacrificed animals, subjected to standard histological staining, and blinded review by a neuropathologist. Blinded analysis revealed all treated animals analyzed had no residual tumor. Some minimal areas of necrosis were identified in a few specimens, with a mild rim of surrounding gliosis and scattered CD68+ macrophages.

To confirm these results were not specific to the model being utilized and to lend further support for potential clinical activity, the experiments were repeated in a 251U intracranial xenograft model. This model is known for its more pathognomonic features on histology such as pseudopalisading necrosis, as well as its highly invasive nature. In these experiments, control animals had a median survival of 30 days post inoculation with all animals succumbing to the tumor by day 34. In contrast, all treated animals survived through 90 days post treatment with no evidence of neurologic compromise. These animals maintained their weight and had no further luciferase activity on non-invasive imaging, suggestive of complete tumor eradication. No residual tumor was observed in treated animals upon sacrifice either grossly or on histologic sectioning, and gliosis was minimal. This further supported the efficacy of $^{186}$RNL for treatment of glioblastoma, and as noted by others (James 2012) were "impressive and provide a reason for optimism regarding this treatment approach for patients with GBM."

### 1.2.2. GLP Safety Assessment

Following preclinical evaluation showing substantial ability of the mammalian CNS to tolerate precisely delivered, high doses of radiation, a preclinical laboratory study was designed and directed by the PI (MPI Study Number 2171-001, IND 116117 Initial IND) and conducted in accordance with the United States Food and Drug Administration (FDA) Good Laboratory Practice (GLP) Regulations, 21 Code of Federal Regulations (CFR) Part 58 at MPI Research (Mattawan, MI), The primary objective of this study was to assess overall toxicity (both local brain and distant organ) of a single dose administration by the intracranial route of the $^{186}$RNL. The secondary objective was to evaluate dosimetry following a single dose administration of $^{186}$RNL.

Three treatment groups of 3 or 5 male and 3 or 5 female beagle dogs underwent a surgical procedure in which a cannula was advanced into the parietal lobe of the brain. The treated animals were administered the test article at respective absorbed dose levels of 1, 3.5, and 6 mCi $^{186}$RNL. One additional group of 5 animals/sex served as the control and received "blank" nanoliposomes as the control article. The control or test article was administered to all groups via a single

intracranial injection once on Day 0, at a dose volume of 0.260 mL. Three animals/sex/group were designated for a 24-hour recovery period. The remaining 2 animals/sex at 0 and 6 mCi were designated for a 14-day recovery period.

Observations for morbidity, mortality, injury, and food and water intake were conducted twice daily for all animals. Clinical observations were conducted daily beginning on Day -7 and throughout the study. Body weights were measured and recorded within 24 hours prior to dosing, weekly during the study, and on the day of necropsy. Food consumption was measured and recorded daily (beginning on Day -2) for all 14-day recovery animals. Ophthalmoscopic, physical, and electrocardiographic examinations were conducted on all animals, pretest and 12 to 24 hours post dose, as well as on all 14-day recovery animals prior to necropsy. Gamma Camera Imaging was conducted on all treated animals 1 and 24 hours post dose, and 3- and 5-days post dose. Blood and urine samples for clinical pathology evaluations were collected, as well as bone marrow smears from all surviving animals at necropsy. Blood samples for determination of the whole blood scintillation counts were collected from all animals at 5 minutes, 1, 5-, 24-, 72-, and 96-hours post-dose. At study termination, necropsy examinations were performed, organ weights were recorded, and the brain microscopically examined.

Intracranial administration of 1, 3.5, or 6 mCi $^{186}$RNL or control article produced no significant test article-related pathologic changes systemically or in the brains of dogs at 24 hours or 14 days. Macroscopic and microscopic changes seen were judged to be a result of the dosing procedure. Based on these data the no adverse effect limit (NOAEL) as related to brain pathology was determined to be 6 mCi $^{186}$RNL in the beagle dog as a single infusion when assessed at 24 hours and 14 days. Additionally, these large animal toxicology studies provided needed support for the safety of the BrainLab catheters in support of subsequent FDA clearance.

## 1.3. Clinical Data

### 1.3.1. Phase 1 First-in-Human Study in Adults with Recurrent Glioma

$^{186}$RNL has been under investigation in an ongoing, first-in-human, open-label, Phase 1 study investigating dose escalation and other delivery parameters (i.e., number of catheters (1-4), infusion rates, volumes, and concentrations) to determine the maximum tolerated dose (MTD), maximum feasible dose (MFD), safety, and efficacy of $^{186}$RNL in recurrent glioma. The study used a modified 3x3 Fibonacci dose escalation, followed by an expansion at the non-DLT/MTD/MFD to determine efficacy. The starting administered dose was 1 mCi in a volume of 0.66 mL. $^{186}$RNL is infused direction into the tumor via Convection Enhanced Deliver (CED).

Male and female study participants at least 18 years of age with recurrent glioma (as defined by the 2021 WHO classification of Tumors of the Central Nervous System) are included in the study. Enrolled patients must show progression by Radiographic Assessment in Neuro-Oncology (RANO) criteria following standard treatment options with known survival benefit (e.g., surgery, temozolomide, radiation, and tumor treating fields), unless medically unable or unwilling to follow standard treatment options for any recurrence.

At the time of this amendment, 28 study participants have been consented and screened for the

Phase 1 study. Of these, 24 have been enrolled in the Phase 1 study.

### 1.3.2. Dose Escalation

Phase 1 has a total of 8 Cohorts (Table 1), with each Cohort varying in dose, volume, concentration, and/or infusion rate. At the time of this amendment, there have been 24 patients over 7 Cohorts (1-7) treated with $^{186}$RNL.

| Cohort | Infused Volume (mL) | Total $^{186}$RNL Activity | Concentration (mCi/mL) | Average Absorbed Dose (Gy) | Status |
|---|---|---|---|---|---|
| 1 | 0.66 | 1.0 | 1.5 | 198 | Enrolling Cohort 8 (n = 24 subjects) |
| 2 | 1.32 | 2.0 | 1.5 | 122 | |
| 3 | 2.64 | 4.0 | 1.5 | 243 | |
| 4 | 5.28 | 8.0 | 1.5 | 171 | |
| 5 | 5.28 | 13.4 | 2.5 | 423 | |
| 6a | 8.80 | 22.3 | 2.5 | 287 | |
| 6b* | 8.80 | 22.3 | 2.5 | 584 | |
| 7 | 12.3 | 31.2 | 2.5 | In analysis | |
| 8 | 16.34 | 41.5 | 2.5 | TBD | |

Table 1**.** Phase 1 Dose Escalation plan for $^{186}$RNL. Cohort 6b utilized the same volume and dose as Cohort 6a but with an increase in maximum flow rate to 20 microliters/minute.

As of the time of this amendment, the following 7 Cohorts have been completed:

- Cohort 1, 3 subjects: 1.0 mCi in 0.66 mL infusate
- Cohort 2, 3 subjects: 2.0 mCi in 1.32 mL infusate
- Cohort 3, 3 subjects: 4.0 mCi in 2.64 mL infusate
- Cohort 4, 3 subjects: 8.0 mCi in 5.28 mL infusate
- Cohort 5, 3 subjects: 13.4 mCi in 5.28 mL infusate
- Cohort 6a, 3 subjects: 22.3 mCi in 8.80 mL infusate
- Cohort 6b, 3 subjects: 22.3 mCi in 8.80 mL infusate (flow rate increased to maximum of 20 uL/min)
- Cohort 7, 3 subjects: 31.2 mCi in 12.3 mL infusate

Cohort 8 (3 subjects, 41.5 mCi in 16.34 infusate) is in progress.

As of the time of this amendment, a total of 4 participants were ongoing (active phase of trial and in overall survival (OS) follow-up contact).

### 1.3.3. Primary Endpoints

The primary objective of the Phase 1 study is to determine the safety, tolerability, and MTD/MFD of $^{186}$RNL administered by CED in the treatment of recurrent gliomas. There have been no clinical Dose Limiting Toxicities (DLT) in the 24 patients treated with $^{186}$RNL to date. Most AEs are mild to moderate (Grade 1 or 2) and, except for scalp discomfort due to CED catheter placement,

unrelated to $^{186}$RNL. SAEs were Grade 3 or less and unrelated except for one possible SAE (vasogenic cerebral edema) due to too rapid glucocorticoid medication taper which was promptly resolved with medication adjustment. In addition to exhibiting a favorable safety profile, the early data provides compelling preliminary clinical evidence of biological response and potential efficacy in the tumor sizes (up to ~20-22 cm$^3$) being evaluated.

### 1.3.4. Secondary Endpoints

The determination of objective response and survival are secondary endpoints for this Phase 1 study.

Across the first 23 study participants, percent tumor volume in the treatment volume (tumor coverage) and the absorbed dose of radiation to the tumor (tumor dose) appear to have the best correlation to overall survival (OS). Relatedly, prior treatment with bevacizumab, correlates with poor $^{186}$RNL convection to the tumor and therefore low tumor coverage and dose. Specifically, 5 patients total were enrolled in Cohorts 2, 3, and 4 that had prior bevacizumab. Only 1 of 5 patients receiving prior bevacizumab had tumor coverage greater than 60% and tumor dose of greater than 60 Gy. The mean OS in this group receiving <100Gy was ~22 weeks. Subsequently, prior treatment with bevacizumab has been used as an exclusion criterion.

### 1.3.5. Dosimetry

Radiation dosimetry was evaluated by SPECT/CT imaging following administration of $^{186}$RNL. In the Cohorts completed to date (1-7), the absorbed radiation dose following treatment with $^{186}$RNL was highly specific to the tumor compared with the absorbed dose in other tissues including brain and in the total body. Whole body and normal organ dosimetry is low without risk for radiation toxicity. As no dose limiting toxicities have been observed, the Sponsor plans to continue to dose escalate in its current Phase 1 trial (Cohort 8) to explore treatment of larger tumors and further alteration of key delivery parameters such as catheter number and increased flow rate.

Each enrolled patient underwent post treatment evaluation of radiation dosimetry. This analysis included assessment of the average absorbed dose of radiation to the tumor, percent coverage of the tumor with radiation (% TuV/TrV), and spatio-temporal assessment of the radiation/tumor relationship. Based on this analysis, the Sponsor could determine the relative volume and location of radiation in the brain at up to 5 days post-treatment and its relationship to the imagined tumor mass. Over the course of the study, average absorbed dose to the tumor and % TuV/TrV increased. Thus far, the maximum absorbed dose to the tumor is 740 Gy and % TuV/TrV > 70% is the norm.

### 1.3.6. Retreatment

In addition to continuing enrollment in the Phase 1 trial through Cohort 8, the Sponsor submitted an IND Amendment (SN0034) to propose a new protocol (to be conducted in parallel with the ongoing Phase 1 and Phase 2 trial) to include patients who reoccur after enrollment in the ReSPECT study (Study #2021-GM-001). No patients have met enrollment criteria to be enrolled for retreatment at this time.

### 1.4. Other Clinical Studies

In addition to published results with $^{186}$RNL, others have performed evaluation of non-encapsulated $^{186}$Re. While these studies are lacking in relation to $^{186}$RNL from a drug control standpoint, as non-encapsulated drug is not significantly retained, they do support the use of non-encapsulated $^{186}$Re. This includes the following cases:

- The management of intramedullary cystic pilocytic astrocytoma with $^{186}$Re intracavitary irradiation in a 22-year-old male patient, in which interstitial intracavitary rhenium brachytherapy of recurrent spinal cord cystic astrocytomas achieved excellent stabilization of the cyst with minor side effects and dramatic improvement of neurological deficits (Colnat-Coulbois et al. 2010).
- The combination treatment for pilocytic astrocytoma by stereotaxic radiosurgery and endocavitary radiotherapy in a 7-year-old male patient. In this report, following multi-beam irradiation, the increase in size of the cyst imposed further intracavitary radiation using $^{186}$Re to deliver 400 Gy to the cyst wall. After a period of intense cerebral edema, resolutive with steroid treatment, progressive cyst disappearance and mural nodule retraction were observed. A PET scan performed 3 years after this treatment revealed no metabolic activity in the persistent mural nodule and the patient remained asymptomatic (Proust et al. 1998).

## 2. RATIONALE FOR THE STUDY

Rhenium-186 Nanoliposome ($^{186}$RNL) is fully retained within its liposome encapsulation shell allowing significant tumor/tissue retention by locoregional direct administration. Once delivered to the target tissue, it emits therapeutic beta particles and induces absorbed radiation dose dependent cell damage including double-stranded tumor DNA lethal destruction. An optimal half-life (<~90 hours) for a therapeutic isotope allows for logistical flexibility while, in parallel, providing high absorbed radiation doses to the tumor. The average tissue path length of ~1.8-2 mm for the β-decay particle energy deposition is similarly ideal for intra-tumoral distribution heterogeneity without toxicity or significant normal tissues exposure. Once administered into the target tissue, retention is facilitated through its liposomal encapsulation. Furthermore, Re-186's decay also has simultaneous gamma photons (10% occurrence) allowing for real time imaging of $^{186}$RNL's *in vivo* distribution, during and after administration where this durable retention has been objectively confirmed. Importantly, this real time monitoring of therapeutic infusion can easily be performed on standard nuclear imaging equipment that are available in routine medical practice and facilitating targeted delivery and dosimetry.

By comparison, external beam radiation therapy (EBRT) must go through normal tissue before and/or after reaching the tumor, limiting the amount of therapeutic radiation safely delivered to the tumor, and must be delivered on multiple days over many weeks. For these critical limitations, toxicity, and EBRT's repetitive delivery over many weeks, $^{186}$RNL holds significant potential to provide an important advance in tumor treatment and patient management. The Sponsor's preclinical and ongoing clinical program has no observed/reported DLTs and no clinically important AEs or SAEs, coupled with a very well tolerated $^{186}$RNL administration. This rationale supports continuing the dose escalation study for the largest recurrent gliomas, as described for the Phase 1 study, and taking the non-DLT Cohort 6 dose from the ongoing Phase 1 dose escalation

clinical study and advancing into a Phase 2 study to determine the efficacy and safety of $^{186}$Rhenium Nanoliposomes ($^{186}$RNL) in recurrent glioma.

### 2.1. Rationale for selection of the subject population

There are no effective established treatments for high grade glioma available after progression on standard therapy, and to date only radiation, alkylating chemotherapy, and tumor treating fields have improved survival. Effective treatment options have not progressed in decades, with glioblastoma having dire 5-year survival statistics. These patients invariably recur and have a dismal prognosis with all patients succumbing to their disease. In this study, patients with recurrent glioma (as defined by the 2021 WHO CNS5) will be eligible for enrollment. Patients will be further restricted to bevacizumab naïve to allow for survival comparison with historical controls.

### 2.2. Study Compliance

This study will be conducted in compliance with this protocol, the principles of Good Clinical Practices, and applicable regulations.

## 3. OBJECTIVES OF THE STUDY

### 3.1. Primary Objectives

#### 3.1.1. Phase 1

To determine the maximum tolerated dose of $^{186}$RNL by convection enhanced delivery (CED) at the time of planned stereotactic biopsy, when necessary, as standard of care.

#### 3.1.2. Phase 2

To assess overall survival (OS) following $^{186}$RNL administration by convection enhanced delivery (CED) in patients with recurrent glioma.

### 3.2. Secondary Objectives

#### 3.2.1. Phase 1

1. To assess the safety of single dose $^{186}$RNL by CED.
2. To assess the dose distribution of $^{186}$RNL by CED.
3. To determine the overall response rate by Radiographic Assessment in Neuro-Oncology (RANO) criteria following $^{186}$RNL treatment.
4. To determine disease specific progression-free survival after $^{186}$RNL treatment.

#### 3.2.2. Phase 2

1. To assess the safety and tolerability of $^{186}$RNL by the National Cancer Institute (NCI) Common Terminology Criteria for Adverse Events (CTCAE) v4.0 criteria. Safety and tolerability will

be defined by the percent of participants experiencing ≥ Grade 3 AE/SAE.
2. To determine the objective response rate (ORR) from the date of complete or partial response or Serious Treatment-Emergent Adverse Events (Safety and Tolerability) *up to 3 years*.
3. To determine progression free survival at 6 months (PFS-6) as measured from the initiation of study treatment until the date of first documented progression by modified RANO criteria (recognizing the potential of pseudo progression significantly complicating the use of the RANO criteria) or date of death from any cause *up to 6 months*.
4. To determine progression free survival (PFS) as measured from the initiation of study treatment until the date of first documented progression by modified RANO criteria (recognizing the potential of pseudo progression significantly complicating the use of the RANO criteria) where progression is defined as >25% in the sum of products of the perpendicular diameters of CE lesions, evidence of new lesion(s), or date of death from any cause, whichever comes first, *up to 3 years*.
5. Evaluate assessing quality of life using a Quality-of-Life Questionnaire (developed in collaboration with established glioblastoma patient advocacy groups).

# 4. STUDY DESIGN AND METHODS

## 4.1. Phase 1

### 4.1.1. Overview

This is a multi-center, sequential cohort, open-label, volume and dose escalation study of the safety, tolerability, and distribution of $^{186}$RNL given by convection enhanced delivery to patients with recurrent glioma (as defined by WHO CNS5) after standard surgical, radiation, and/or chemotherapy treatment.

At the time of this amendment, there have been 24 patients over 7 Cohorts (1-7) treated with $^{186}$RNL. The initial study parameters are listed below, and the resulting data presented in Section 1.3, *Clinical Data*.

The study uses a modified Fibonacci dose escalation, followed by an expansion at the MTD to determine efficacy. The starting absorbed dose is 1 mCi in a volume of 0.660 mL. This dose and volume were selected after careful consideration by the investigators based on the following:

1. No lethal or maximum tolerated dose could be established in our rodent studies; animals treated with absorbed doses up to 2000 Gy showed no signs of neurologic dysfunction or compromise (Phillips et al. 2012).
2. The toxicity of $^{186}$RNL was evaluated in beagle dogs following single dose administration by the intracranial route. Intracranial administration of up to 6 mCi $^{186}$RNL resulted in no major significant adverse effects. No effects were seen in brain pathology either macroscopically or microscopically that were deemed related to the dose of test article. The NOAEL of the GLP beagle study was therefore deemed to be an administered dose of 6 mCi, corresponding to an absorbed dose of 360 Gy.
3. The starting infused volume of 0.660 mL will leave a significant safety margin when considering the possibility of mass effect.

4. Patients in the first cohort will be limited to an infused volume of 0.660 mL. Based on our previous results with the 45% dose retention and 50-hour biological half clearance time (not including physical decay), this treated volume would correspond to an administered dose of 1 mCi or less to achieve an absorbed dose of 60 Gy.
5. Systemically administered $^{186}$Re-hydroxyethylidene diphosphonate (HEDP), commonly used for palliation of bone metastasis, has an MTD of 80 mCi in prostate cancer patients and 65 mCi in breast cancer patients (Lam et al. 2004). This is 80-fold our starting administered dose.
6. Systemically administered $^{186}$Re labeled chimeric monoclonal antibody U36 (which targets CD44) given to patients with metastatic head and neck cancer was found to have an MTD of 27 mCi per square meter of body surface area (Colnot et al. 2000), which corresponds to at least 17-fold higher than our starting administered dose in a patient of normal habitus.
7. Locally administered doses of 30-60 mCi $^{186}$Re-lipiodol to hepatomas was found to be without unacceptable toxicity (Shi et al. 2002), corresponding to 30- to 60-fold activity higher than our starting administered dose.
8. Locally administered $^{186}$Re colloid of 3.6 mCi for an absorbed dose of 1560 Gy to a patient with a pilocytic astrocytoma of the spine was observed without significant toxicity (Colnat-Coulbois et al. 2010), 3.6-fold the starting administered dose.
9. $^{188}$Re-nimotuzumab introduced by non-CED methods into a surgical cavity of patient with glioblastoma produced a maximum tolerable dose of 10 mCi, 10-fold our starting administered dose (Chopra 2004). The calculated absorbed dose was based on the treatment of 100 grams of tissue (markedly larger than treatment volumes used in our application). While the 10 mCi absorbed over this large volume equates to only 20 Gy, if extrapolated to the maximum treatment volumes being explored in this study, they would be equivalent to greater than 400 Gy. Additionally, it should be noted that we cannot directly compare the dose ratio difference between $^{186}$RNL and $^{188}$Re-antibodies, since we do not know the difference in their clearance dynamics and the 2-fold beta energy and path length of $^{188}$Rhenium to $^{186}$Rhenium.

Escalation will be performed by increases in $^{186}$RNL activity (mCi), with the first three escalations achieved by dose doubling followed by a modified Fibonacci dose escalation scheme (i.e., dose increases of 67%, 50%, 40% and 33%) (Table 2). Dose escalation will be a standard 3+3 design, with escalation between Cohorts dependent on the number of observed dose limiting toxicities (DLTs):

For this study, a DLT is defined as:

- Grade 3 or greater acute CNS toxicity attributable to the study intervention which persists for 96 hours or more (see below discussion of delayed events) OR
- Grade 3 or greater non-CNS toxicity which is attributable to the study intervention.

If a DLT is observed in 1 out of 3 patients at a given dose level, up to an additional 3 patients will be enrolled and treated at that dose level.

If 2 out of 3-6 patients at that dose level have DLTs, the dose will be decreased to the previous dose level and up to 3 additional patients will be enrolled at that dose level for a total of 6 patients.

When up to 3 additional patients are added to a given dose level, if 1 of 6 patients has a DLT then

the dose will be increased to the next dose level.

Given the possibility for radiation effects outside of the traditional DLT window, the following consideration will also be given:

- The DLT evaluation period for CNS toxicity will include 90 days between Cohorts. This will not limit accrual within a Cohort, but across successive Cohorts. As an example, the first patient of Cohort 3 cannot be enrolled until the first patient of the Cohort 2 has completed 90 days of CNS toxicity evaluation.
- If a patient within a Cohort experiences CNS toxicity that would be defined as dose limiting, the entire Cohort must complete 90 days evaluation before the successive Cohort may commence.
- For determination of the Phase 2, 6 months' total time must elapse following the final Cohort for evaluation of CNS toxicity before the Phase 2 portion may begin.

A safety review was performed including review of all toxicities prior to escalating above 8.0 mCi; these results were communicated to the FDA in Amendment SN0013. In addition, in IND Amendment SN0026 DSMB Reports for all cohorts up to and including Cohort 6 was provided to the FDA. Dose escalation will progress until a maximum tolerated dose (MTD) is reached. The MTD is defined as the highest dose-level at which ≤1 of 6 patients in a cohort develop an emergent DLT.

| Cohort | Infused Volume (mL) | Total 186 RNL Activity | Distribution Volume (mL)* | Treatment Field Volume (mL)* | % Total Activity Increase |
|---|---|---|---|---|---|
| 1 | 0.66 | 1.0 | 2.64 | 5.0 | n/a |
| 2 | 1.32 | 2.0 | 5.28 | 8.8 | 100% |
| 3 | 2.64 | 4.0 | 10.5 | 16.0 | 100% |
| 4 | 5.28 | 8.0 | 13.2 | 19.4 | 100% |
| 5 | 5.28 | 13.4 | 13.2 | 19.4 | 67% |
| 6a | 8.80 | 22.3 | 19.4 | 27.2 | 50% |
| 6b* | 8.80 | 22.3 | 19.4 | 27.2 | 50% |
| 7 | 12.3 | 31.2 | 26.4 | 35.9 | 40% |
| 8 | 16.34 | 41.5 | 33.3 | 44.3 | 33% |

Table 2: Modified Fibonacci dose escalation scheme. Cohort 6b utilized the same volume and dose as Cohort 6a but with an increase maximum flow rate to 20 microliters/min.

Cohorts in grey are completed as of the time of this amendment (1-7).

### 4.1.2. Subject Assignment Methods

This is an open-label study and all the Phase 1 patients enrolled will receive $^{186}$RNL. Once an eligible subject is identified and consented, all eligibility studies will be performed prior to treatment planning. A 28-day period is required for toxicity assessment following study treatment of the first patient in each Cohort prior to enrollment of additional patients in the Cohort. Additionally, 90 days is required for toxicity assessment following study treatment of the first

patient in each Cohort prior to enrollment of the same patient in the subsequent cohort.

#### 4.1.3. Statistical Analysis

The actual sample size depends on the unknown dose-toxicity profile of the treatment. Although the traditional 3+3 design commonly used in oncology Phase 1 trials is thought to produce a 33% toxicity rate at the MTD, it has been demonstrated that it is closer to 20-25% for the majority of the dose-toxicity curves considered (Kang and Ahn 2002).

The number of subjects screened, the number of screen failures by reason, the number enrolled, and the number completing $^{186}$RNL treatment will be tabulated. The analysis of the dose escalation results will be descriptive. All adverse events experienced at each dose level will be tabulated. The MTD and the percent and 95% confidence interval of subjects in the expansion cohort experiencing DLT will be reported. The distribution of time to progression among the patients who complete $^{186}$RNL treatment will be summarized with a Kaplan Meier curve. All statistical testing will be two-sided with a significance level of 5%. Analyses will be performed by a statistician.

### 4.2. Phase 2

#### 4.2.1. Overview

This is a multicenter, single arm, non-blinded, prospective study of $^{186}$RNL by Convection Enhanced Delivery in patients experiencing a recurrence following standard multimodal therapy for glioma, as defined by the fifth edition of the World Health Organization's (WHO) Classification of Tumors of the Central Nervous System (CNS) (2021 WHO CNS5). Tumor volume will be limited to ≤20 cm$^3$.

#### 4.2.2. Clinical Endpoints

The primary endpoint will be to assess overall survival (OS). Overall survival will be defined as the time (in months) between the first day of study treatment until death from any cause. Any patient not known to have died at the time of final analysis will be censored based on the last recorded date on which the patient was known to be alive. The secondary endpoints include assessment of the safety and tolerability of $^{186}$RNL, the objective response rate (ORR), progression free survival at 6 months (PFS-6), progression free survival (PFS), and Quality of Life.

#### 4.2.3. Therapy

Patients will receive a single dose treatment of 22.3 mCi (total $^{186}$RNL activity) at a concentration of 2.5 mCi/mL and 8.8 mL total volume. The dose will be infused utilizing one to four catheters based upon PI and surgeon's assessment of tumor size, shape, location, and results of the treatment simulation. The treatment simulation will be performed in iPlan Flow and iterative modulation to the simulation algorithm will be performed as needed.

#### 4.2.4. Disease Assessment

Study subjects will be routinely assessed by MRI (standard of care) until disease progression according to RANO criteria. There will be an additional follow up for overall survival up to a period of 36 months (3 years). Radiographic response to study drug ($^{186}$RNL) will be determined in comparison to pretreatment baseline. The smallest tumor measurement will be used for determination of progression. If subsequent imaging studies demonstrate that progression has occurred, the date of progression should be the date of the scan at which this issue was first raised. All measurable and non-measurable lesions will be assessed using the same techniques as at baseline on the same MRI scanner, or at minimum with the same magnet strength, to reduce difficulties in interpreting changes. Assessment of response by RANO criteria will be used, recognizing the potential of pseudo-progression significantly complicating the use of the RANO criteria.

#### 4.2.5. Imaging Parameters

All imaging time points will be performed as per the recently established international brain tumor imaging protocol (BTIP) with recommended sequences and parameters. This includes pre- and post-contrast 3D (volumetric) inversion recovery gradient recalled echo (IR-GRE) images with less than 1.5-mm isotropic resolution, which allows for both bidimensional and volumetric measurements of enhancing tumor. Investigator assessments will be independently confirmed and supplemented with volumetric assessment retrospectively. Additionally, due to the concern of possible pseudo-progression (radiation treatment effect resulting in increased enhancement), we will utilize delayed contrast extravasation imaging at each time point to generate Tumor Response Assessment Maps (TRAMs). Post contrast 3D T1 MRI is obtained within 5 min and greater than 1 hr. post contrast injection, followed by rigid/elastic registration and intensity variation corrections, and creation of subtraction maps of early images from the late images. Histological validation confirmed that regions of contrast agent clearance in the TRAMs >1 hr. post-contrast injection represent active tumor, while regions of contrast accumulation represent nontumor tissues with 100% sensitivity and 92% positive predictive value to active tumor.

#### 4.2.6. Statistical Analysis

This phase of the study will include up to 34 experimental subjects with an accrual interval of 36 months. Based on historical clinical glioblastoma data, the median overall survival (OS) of control patients (e.g., salvage therapy with lomustine or bevacizumab was approximately 8 months. If the true median OS in the historical control group and the experimental treatment group treated at the MTD are 8 months and 10.4 months, then assuming a two-parameter Weibull model, two-sided one-sample log-rank testing, and alpha=0.05, this study will attain a power of 80% for testing Ho:SE(t)=SH(t) with n=34 subjects, where SE(t) and SH(t) are the experimental and historical survival distribution functions (PASS Version 11, NCSS, Kaysville, Utah).

The numbers of patients screened, screen failures by reason, the number enrolled and completing the study. The distribution of time death from any cause will be summarized with a Kaplan-Meier curve with an overlaid Weibull model of the historical overall survival function and the p-value for a one-sample two-sided log-rank test comparing the experimental and historical overall

survival distributions will be shown. Time to progression will be summarized with a Kaplan Meier curve and compared with a historical control curve with a one-sample two-sided log-rank test. A one-sample O'Brien-Fleming group-sequential analysis for efficacy regarding overall survival with one interim look at information time=0.5 will be carried out. Futility will be indicated if the boundary is not crossed at the first look and if the conditional power is less than 20%. The decision to stop the trial or continue will be made by the Data Safety Monitoring Committee. Adverse events will be tabulated. Analyses will be performed by a statistician.

# 5. PATIENT POPULATION

## 5.1. Inclusion Criteria

1. At least 18 years of age.
2. Ability to understand the purposes and risks of the study and has signed a written informed consent form approved by the investigator's IRB/Ethics Committee.
3. Histologically confirmed Grade III/IV recurrent Glioma (following 2021 WHO CNS5 glioma nomenclature, e.g., Astrocytoma, IDH-mutant grade 3 or 4; Glioblastoma, IDH-wildtype grade 4).
4. Progression by RANO criteria or other clinically accepted neurooncology evaluation, following standard treatment options with known survival benefit for any recurrence (e.g., surgery, temozolomide, radiation, and tumor treating fields). Patient may be included in study if medically unable or unwilling to follow standard treatment options for any recurrence.
5. Patients who receive treatment with antiepileptic medications must have a two-week history of stable dose of antiepileptic without seizures prior to study start (dosing).
6. Patients with corticosteroid requirements to control cerebral edema must be maintained at a stable or decreasing dose for a minimum of two weeks without progression of clinical symptoms (Section 6.3.2.7) prior to study start (dosing).
7. Patients with Grade III/IV Glioma (following 2021 WHO CNS5 glioma nomenclature, e.g., Astrocytoma, IDH-mutant grade 3 or 4; Glioblastoma, IDH-wildtype grade 4) which falls within the treatment field volume.
8. ECOG performance status of 0 to 2; Karnofsky Performance Status ≥ 60.
9. Life expectancy of at least 2 months.
10. Acceptable liver function:
    - Bilirubin ≤ 1.5 times upper limit of normal
    - AST (SGOT) and ALT (SGPT) ≤ 3.0 times upper limit of normal (ULN)
11. Acceptable renal function:
    - Serum creatinine ≤1.5xULN
12. Acceptable hematologic status (without hematologic support):
    - ANC ≥1000 cells/uL
    - Platelet count ≥100,000/uL
    - Hemoglobin ≥9.0 g/dL
13. All women of childbearing potential must have a negative serum pregnancy test and male and female subjects must agree to use effective means of contraception (for example, surgical sterilization or the use of barrier contraception with either a condom or diaphragm in conjunction with spermicidal gel or an IUD) with their partner from entry into the study through 6 months after the last dose.

### 5.2. Exclusion Criteria

1. The subject has evidence of acute intracranial or intratumoral hemorrhage either by magnetic resonance imaging (MRI) or computerized tomography (CT) scan. Subjects with resolving hemorrhage changes, punctate hemorrhage, or hemosiderin are eligible.
2. The subject is unable or contraindicated to undergo MRI scan (e.g., has pacemaker or medically unstable).
3. The subject has not recovered to CTCAE v4.0 Grade ≤1 from AEs (except alopecia, anemia, and lymphopenia) due to antineoplastic agents, investigational drugs, or other medications that were administered prior to study.
4. The subject is pregnant or breast-feeding.
5. The subject has serious intercurrent illness, as determined by the treating physician, which would compromise either patient safety or study outcomes such as:
   - hypertension (two or more blood pressure readings performed at screening of >150 mmHg systolic or >100 mmHg diastolic) despite optimal treatment
   - active medically significant infection unresponsive to antibiotics (e.g., non-healing wound, ulcer), uncontrolled systemic infection, or bone fracture
   - clinically significant cardiac arrhythmias not controlled by appropriate medications
   - untreated hypothyroidism
   - symptomatic congestive heart failure or unstable angina pectoris within 3 months prior to study drug
   - myocardial infarction, stroke, or transient ischemic attack within 6 months prior to study drug
   - known active malignancy (other than glioma) except non-melanoma skin cancer or carcinoma in-situ in the cervix unless PI determines it would not impact patient safety or efficacy determinations
6. The subject has inherited bleeding diathesis or coagulopathy with the risk of bleeding.
7. The subject has received any of the following prior anticancer therapy:
   - Prior treatment with Bevacizumab
   - Non-standard radiation therapy such as brachytherapy, systemic radioisotope therapy, or intra-operative radiotherapy (IORT) to the target site
   - Radiation therapy within 12 weeks of screening
   - Systemic therapy (including investigational agents and small-molecule kinase inhibitors) or non-cytotoxic hormonal therapy (e.g., tamoxifen) within 14 days or 5 half-lives, whichever is shorter, prior to study start (dosing)
   - Biologic agents (antibodies, immune modulators, vaccines, cytokines) within 21 days prior to study start (dosing)
   - Nitrosoureas or mitomycin C within 42 days, or metronomic/protracted low- dose chemotherapy within 14 days, or other cytotoxic chemotherapy within 28 days, prior to study start (dosing)
   - Prior treatment with carmustine wafers
   - Patients who are currently receiving any other investigational agents and/or who have received an investigational agent in 28 days prior to study start (dosing)
8. Multifocal progression or involvement of the leptomeninges.
9. Psychiatric illness/social situations that would limit compliance with the study requirements.
10. Infratentorial disease.

11. The subject has a tumor located within 1-2 cm of a ventricle AND it is determined by the surgeon, PI, and sponsor to be a risk for drug extravasation to the subarachnoid space if given catheter placement and drug administration.
12. Phase 2 only: The subject should have a tumor volume of ≤20 $cm^3$ to be included in the Phase 2 portion of the study. Subjects with tumor volumes of greater than 20 $cm^3$ are excluded from the Phase 2 portion of the study.

# 6. PROCEDURES

## 6.1. Summary

For both the Phase 1 and 2, subjects are expected to participate for up to 13-14 weeks with continued follow up for survival until three years after the first dose of study drug. Please refer to Appendix A, Schedule of Events, for an overview of the study assessments. Subjects who withdraw from the study before all follow-up procedures have been performed will be managed and documented as described in Section 8, Removing Subjects from the Study.

A summary of visits and clinical procedures is found in Appendix A, Schedule of Events. The total duration of the **active** part of the study for each subject will be 13-14 weeks, divided as follows:

- Up to 5 weeks pre-dose (screening period)
- Up to 8-9 weeks for CED insertion, dosing, recovery, and toxicity and disease assessment.

After Day 56 (+/- 3 days), the subject will be followed by their physician and / or the PI following standard of care.

An End of Study Update (virtual or in person) will be completed (within 2 weeks if possible, depending on the subject's condition and other scheduling circumstances) after confirmation of disease progression. If pseudo-progression is suspected, the patient may remain on study until a confirmatory scan(s) or other objective diagnostic procedure has been performed to confirm pseudo-progression or disease progression (as per the modified RANO or WHO CNS5 2021 criteria in Appendix C).

When a subject has completed the End of Study Update, they and/or a family member and/or PI will be contacted for survival information every 3 months until three years from first dose.

Scheduling will recognize flexibility for the subject's safety and medical condition, holidays, and other scheduling considerations. However, the exact timing of data acquisition will be required.

## 6.2. Screening

All screening procedures must be done within 35 days of presurgical Day -1, unless otherwise specified.

### 6.2.1. Informed Consent

During screening, candidates for the study will be fully informed about the nature of the study and possible risks and will receive a copy of the Informed Consent form for review. Additionally, candidates will be asked for permission to use protected health information (in accordance with the Health Insurance Portability and Accountability Act or HIPAA). Candidates must read the Informed Consent form (or confirm it was read and understood) and sign the document in the presence of a member of the study team after all patient or family questions have been answered. Further procedures may begin after the Informed Consent form has been signed. The original signed Informed Consent form will be retained by the study site and a copy will be given to the candidate. Candidates will be evaluated for entry into the study according to the stated inclusion and exclusion criteria (Section 5, Patient Population). The study team will evaluate the results of all examinations, including clinical laboratory tests, and will determine each candidate's suitability for the study. The study team must know the baseline results before enrollment. The pregnancy test for females of reproductive potential must be negative for those subjects to proceed to enrollment.

Refusal to sign informed consent and permission excludes an individual from the study.

### 6.2.2. Procedures

The following will be performed to establish each candidate's general health and qualifications for possible enrollment into the study, as outlined by the Schedule of Events (Appendix A):

- Review inclusion and exclusion criteria (Section 5, Patient Population).
- Record medical and surgical history, including cancer history (histology of primary tumor (including degree of differentiation), date of cancer diagnosis, types, and dates of prior anti-tumor therapy (including surgery, radiation therapy, systemic therapy), and date of most recent disease progression).
- Perform a complete physical examination.
- Record blood pressure (BP), heart rate (HR), respiratory rate (RR), and temperature measurements.
- Record height and weight.
- Assess Eastern Cooperative Oncology Group (ECOG) Performance Status score (Appendix B, Eastern Cooperative Oncology Group Performance Status Scale) and Karnofsky Performance Status score.
- Obtain a baseline ECG to assess for cardiac arrhythmias or evidence of recent cardiac events.
- Draw blood samples for hematology, chemistry, and coagulation.
- Obtain a urine sample for urinalysis.
- Obtain a blood sample for serum HCG pregnancy test in female subjects of child- bearing potential (all female subjects unless surgically sterilized or at least 1 year post-menopausal).
- Perform tumor assessment with MRI of the brain to determine eligibility.
- Record recent medication history, including vitamins, herbal preparations, blood products,

and other over the counter (OTC) drugs.

All screening and other HIPAA-compliant, de-identified patient history, labs, and medical information relevant to the subject's study enrollment will be provided to the Sponsor (e.g., Chief Medical Officer and/or Medical Monitor and/or designee).

## 6.3. Treatment

### 6.3.1. Evaluations

Efficacy will be assessed based on tumor assessments (objective response rate, progression-free survival, and duration of response) conducted at intervals during the study (Schedule of Events, Appendix A).

### 6.3.2. Procedures

#### 6.3.2.1. Treatment Planning

The characteristics of tumor to be treated, including location, structure, shape, and dimension defined from the most recent MR image, will be reviewed by the PI. If CT images are available, these will also be reviewed and co-analyzed with MR images. The MR and CT images are recommended to have been acquired with contrast enhancement to better visualize the vasculature surrounding and within the tumor. The tumor volume will be calculated by either a) dimension measurement of the MR image at three orthogonal directions by using the following equation:

$$V = \frac{3.14}{6} \; xaxbxc$$

Where a, b, and c are tumor dimensions in the unit of mm. V is tumor volume in the unit of □l.

Preferably, the use of computer-aided software such as iPlan (BrainLab) is used. Following the prescreening MRI (Schedule of Events, Appendix A), the MR images are uploaded into a database for review by the PI or co-PI. Using the iPlan software, the PI or co-PI can determine the number of catheters to be used and the suggested location of catheter for the treatment plan protocol. This software, in conjunction with the BrainLab VarioGuide system, allows the neurosurgeon to navigate the SmartFlow catheter to the tumor under intra-procedural MRI guidance.

After image review and communication among the PI, involved physicians, and medical physicist, the treatment team will finalize the treatment plan protocol. For Phase 1 study, the dose escalation plan will be used (Section 4.1.1, Table 2). For the Phase 2 study, 22.3 mCi (total $^{186}$RNL activity) at a concentration of 2.5 mCi/mL and 8.8 mL total volume will be administered at infusion rates and times outlined in Appendix E. Before patient treatment, the involved physicians and the medical physicist will communicate with one another and discuss all critical parameters of the treatment plan and possible concerns. The final treatment plan for each patient will be approved by all involved physicians and the medical physicist before use.

### 6.3.2.2. $^{186}$RNL Manufacture

The $^{186}$RNL will be labelled under sterile conditions at RadioMedix, Inc (Houston, Texas). RadioMedix is a certified radiopharmaceutical contract manufacturer who manufacturers under cGMP. Complete details of manufacture and quality control of the liposomes and preparation of the $^{186}$RNL are as described in the Master Batch Record (MBR). Drug product will be assayed for endotoxin levels and will be less than 175 EU/dose prior to use. Sterility of drug product will be verified as part of delayed testing along with particle size measurements. Liposomes to be labeled with $^{186}$Re will be tested for particle size distribution, pyrogenicity, and sterility prior to performance of the radiolabeling procedure. The mean particle diameter of the liposomes should be ≤130 nm with ≤0.4 polydispersity index. Drug product will be certified by a Board-Certified Nuclear Pharmacist prior to administration.

### 6.3.2.3. Intraoperative Catheter Placement

Following a standard of care (SoC) stereotactic biopsy procedure for confirmation of disease progression, CED catheter placement will ensue with at least one catheter proceeding along the same needle track as the SoC biopsy, or alternatively may proceed along a second preapproved planned track. Multiple catheters may be used at the treating investigator's discretion based on pretreatment planning to allow for suitable coverage of the tumor.

Catheter placement will need to avoid ependymal surfaces by greater than 0.5 cm as they offer no resistance to fluid flow, and the infusate can flow directly into the ventricular or subarachnoid (SA) space and away from the intended tumor bed target. The ventricles are avoided during placement of the catheter for the same reasons. Furthermore, if the subject has a tumor located within 1-2 cm of a ventricle AND it is determined by the surgeon, PI, and sponsor to be a risk for drug extravasation to the subarachnoid space based on treatment planning catheter placement and $^{186}$RNL drug administration, the study subject will be excluded from the study.

Unless broached, the pia arachnoid layers can resist fluid flow; therefore, when passing the infusing catheter, the deep sulci are avoided (utilizing intraoperative image guidance –direct visualization) to prevent the flow infusate moving into the subarachnoid space and away from the target. The infusing tip should be at least 2.5 cm from the cortical surface. Distal placement of the infusing tip within the tumor is critical to avoid the pressure gradient from the tumor core to periphery to adjacent brain. The catheter will be maintained in place utilizing accompanying bone anchor and head wrap. Postoperative head CT is performed to evaluate for hematoma or pneumocephalus. Once each of the catheters are placed and CT and/or the study team confirms no complications, the subject is taken from post anesthesia recovery to a designated room for recovery.

### 6.3.2.4. Postoperative $^{186}$RNL Administration, Imaging, Biodistribution, and Dosimetry

#### $^{186}$RNL Administration

600 mg supersaturated potassium iodide (SSKI), or at the Investigational Site's SSKI administration dose, is administered by mouth with one glass of water or fruit juice (fruit juice

preferred) on the day prior to $^{186}$RNL infusion. Alternatively, the SSKI can be administered up to the start of $^{186}$RNL infusion, but pre-dosing SSKI administration is preferred.

On the day following surgery, the subject is transferred to the imaging suite for $^{186}$RNL infusion (Day 1). The infusion pump(s) will be connected to an AC wall outlet through an intervening portable back-up battery power source. This portable power source will permit the continuous infusion of $^{186}$RNL during transport of the patient from his/her bed to the SPECT/CT camera for imaging during the infusion. Backup power source may be omitted for pumps with internal battery source that have been checked prior to infusion. The initial infusion will begin at a low rate and increase over time (Appendix E).

#### 6.1.1.1.1. Imaging and Biodistribution

Planar and tomographic (SPECT/CT) images will be collected from all patients using a dual-detector SPECT/CT camera.

A sealed $^{186}$RNL vial with known $^{186}$Re radioactivity (~0.2 ml drug product sample at infused dose concentration, diluted to ~1 ml with PBS buffer) will be positioned next to each patient at each time of image acquisition for *in vivo* radioactivity quantification. A representative image acquisition is presented with the acknowledgement there may be site specific selections due to specific instrumentation requirements. Often, the image acquisition uses low energy high resolution (LEHR) parallel-hole collimators and three energy window image collection mode. The three energy windows are: 1) the primary energy window at 137 keV (energy of $^{186}$Re's g-ray) ±10% (some institutions may use narrower energy range for the primary energy window); 2) a lower $^{186}$Re's g-ray energy window at 118 keV ± 3.5%; and 3) a higher $^{186}$Re's g-ray energy window at 159 keV ± 3.5%. Typically, the total number of projections for SPECT images are ≥36 (18 x 2 for dual detector cameras) while the acquisition time for each projection is ≥40 seconds. The raw images from each energy window will be saved separately. The CT imaging protocol (e.g., kVp, mAs, and reconstruction filter) will be maintained for each patient at the site. The SPECT image reconstruction is determined from the primary energy window images following the image reconstruction protocol commonly used for this energy range and for the collimators. If a three-energy window is not feasible, then it is mandatory to use the primary $^{186}$Re's g-ray 137 kev window.

During $^{186}$RNL infusion, the dynamic images will be acquired via a real time persistence scope for evaluation of focal accumulation of activity in the brain at the assumed tip of the catheter(s). When activity is observed to accumulate focally, the time of the beginning of the focal volume accumulation will be designated as the beginning of the planned therapeutic volume infusion into the brain tumor to correct for dead space in the catheter line. The infusion will proceed as described until just prior to infusion of 20% of the planned therapeutic dose, at which time the patient will be moved to the SPECT/CT camera while the infusion pumps continue to operate, using the portable battery power source as required. Using AC power for the pump is preferred to keep pump battery storage capacity as great as possible. Moving the subject to the SPECT/CT camera will not be required if the nuclear medicine team previously decided to acquire the initial planar dynamic images using the SPECT/CT camera (preferred).

The SPECT/CT imaging will be performed and evaluated for the distribution of the $^{186}$RNL activity, following a pre-set imaging protocol for $^{186}$RNL studies. CT and SPECT images will be rapidly reconstructed and evaluated for location of the infusion within the brain. If the infusion is in the planned location, the infusion will continue until the total planned infusion volume has been administered.

After the 20% infused volume, SPECT/CT images are acquired. Dynamic persistence imaging is not a mandatory requirement, and the subject can be moved to a site away from the cameras until the end of the planned infusion at the discretion of the nuclear medicine and/or study team(s). At the end of the $^{186}$RNL infusion, the infusion line(s) may be disconnected, and the patient will be moved to the SPECT/CT camera for performance of 1) the end of infusion (EOI) whole body (head to toe) AP/PA dual detector planar gamma camera imaging, and 2) the end of infusion (EOI) SPECT/CT imaging of the head and upper neck. Note that the standard $^{186}$Re source should be placed at 3-5 cm from the patient's head for the entire imaging procedure (no overlap from patient head from AP/PA view). At the Nuclear Medicine physician discretion, the order in which the SPECT/CT imaging and planar whole-body imaging are done may be reversed. Recording actual time at the start of acquisition is required.

At 24 hours, 72 hours, 120 hours, and 192 hours post-treatment, the patient will be re-imaged with planar whole-body imaging (also SPECT/CT imaging at 24 hours, 120 hours, and 192 hours). Imaging will allow for flexibility for the subject's safety and medical condition (the exact timing of data acquisition will be required).

**Dosimetry**

The counts of radioactivity in each organ will be measured through drawing regions-of-interest (ROI) around each organ on anterior and posterior planar images. The count (I) in each organ will be averaged from anterior and posterior images using geometric mean method: $I = SQRT(I_1 \times I_2)$.

Where:

$I_1$ is the count of an organ from anterior planar image.
$I_2$ is the count of the organ from posterior planar image.

The total uptake of normal organs and tumors at different time intervals will be computed and reported as percent of injected doses (%ID). The total body $^{186}$Re radioactivity will be computed with the summation of total body radioactivity.

Urinary excretion will be evaluated by collecting samples of all the voided urine during the 48 hrs. post-injection, with flexibility for the subject's safety and medical condition (the exact timing of data acquisition will be required). Volumes and times of all urinations will be recorded and measured in duplicate using a dose calibrator if activity is high or a well counter if counts are decayed and lower and compared to a known standard of activity. The sample count rates will be corrected for decay and expressed as a percentage of administered activity. Urinary excretion will be reported as the total percent of injected doses.

Blood will be evaluated by collecting samples at 0.5, 1, 2, 4, 8, 24, 48, 72, and 120 hrs. following end of infusion (EOI), with flexibility for the subject's safety and medical condition (the exact timing of data acquisition will be required). At each timepoint, two 3mL sodium citrate blood collection tubes will be labeled and, following collection, inverted gently before being sent for processing to the reference laboratory. The amount of radioactivity at the time of shipment is minimal regarding radiation safety, and shipments will be treated as regular biohazard samples in shipment. To ensure appropriate handling with acceptable radiation level as regular biohazard samples, shipments for each package will be processed following the hospital's internal/external radiation safety guidelines. Coordination with Nuclear Medicine, the Study Coordinator, and floor personnel (nurses) is required to ensure all post-dose blood samples are correctly collected.

### 6.3.2.5. Post $^{186}$RNL Infusion Care

Following the administration of the $^{186}$RNL dose, the patient will be moved to a designated area following the hospital's internal/external radiation safety guidelines and the designated area will be monitored as required. The imaging room will be monitored for radioactivity and cleaned following the hospital's internal/external radiation safety guidelines.

The catheter is then removed 24-48 hours following completion of infusion at the direction, timing, and discretion of the study team.

Wipe testing will be performed at the catheter insertion sites on the scalp surface (where catheter insertion occurred) to ensure no activity is leaking from the injection site. If activity is still present, the subject will be held in the hospital until no activity is found to be leaking from this site. When the subject is stable and no activity is leaking from this site of injection, the subject will be discharged from the hospital with precautions determined by the study team and the hospital's internal/external radiation and patient safety guidelines. In the absence of site-specific radiation discharge criteria, suggested study discharge criteria would be the following: At the time of discharge, the dose at 1 meter from the patient will be less than 5 mR/hr. (or may be expressed as 5 mrem/hr. depending on the RSO's procedure) as per standard institutional discharge criteria for radioactivity administration (for example, in thyroid cancer patients treated with $^{131}$I). It is important to note that $^{186}$Re only has a ~10% gamma emission and the beta-particles emitted by $^{186}$Re travel less than 2-3 mm in tissue.

### 6.3.2.6. Outpatient Care

The subject will be monitored in the hospital, based on study team recommendations, often for approximately 48 hours. Ultimate patient management is the responsibility of and at the discretion of the PI and physician of record to ensure medically required safety and comfort. If the subject is considered stable by the study team and following no activity leaking from the scalp site, they will be discharged from the hospital as per routine for subjects undergoing stereotactic brain biopsy or CED insertion. Following discharge, the subject will return for imaging according to the Schedule of Events (Appendix A) on an out-patient basis. Likewise, post-treatment evaluations will be done according to the Schedule of Events (Appendix A). The minimum assessment period for patient tolerability and adverse events is 56 +/3 days from CED insertion and $^{186}$RNL administration (Schedule of Events, Appendix A).

The following will be done in all subjects at each post-treatment evaluation, as outlined in the Schedule of Events (Appendix A):

- Medical and surgical history since screening recorded
- Concomitant medications for previous 14 days recorded
- AEs since last visit recorded
- Weight and vital signs recorded
- Detailed physical exam including ECOG performance and neurologic assessment performed and recorded
- Blood samples for hematology and chemistry drawn
- MRI 28 days (+/- 1) and 56 days (+/- 3 days) from treatment with tumor measurements

Scheduling will recognize flexibility for the subject's safety and medical condition, holidays, and other scheduling considerations. However, the exact timing of data acquisition will be required.

#### 6.3.2.7. Use of Corticosteroids

Prophylactic use of corticosteroids is discouraged as the toxicity profile of $^{186}$RNL is not yet known. While treatment related edema is a known complication of external beam radiation, including that associated with early radiation injury or radiation necrosis, the clinical experience with brachytherapy in the brain is limited. Further, patients will be monitored closely for toxicity. This approach is consistent with previously conducted trials with ablative radiotherapy, including the Radiation Therapy Oncology Group's study 90-05 (Shaw et al. 2000). Patients in this study received stereotactic external beam irradiation for recurrent primary brain tumors or CNS metastases. In this study it was notable that 73% of patients that were not on steroids at the time of treatment with radiosurgery were able to remain off steroids following treatment. Nevertheless, use of corticosteroids to treat any clinical symptoms which are felt to be the consequence of cerebral edema is STRONGLY encouraged and critical to patient safety. The total dose must be recorded pre-treatment, and at the time of each evaluation. Steroids will be used in the smallest dose that will afford the patient satisfactory neurologic function and the best possible quality of life.

## 6.4. End of Study, Early Study Termination, and Survival Follow-up

### 6.4.1. End of Study or Early Study Termination

The following will be done at the End of Study visit (Day 56 (+/- 3 days) or Early Termination Visit as outlined in the Schedule of Events (Appendix A):

- Record medical and surgical history, including cancer history (histology of primary tumor (including degree of differentiation), date of cancer diagnosis, types, and dates of prior anti-tumor therapy (including surgery, radiation therapy, systemic therapy), and date of most recent disease progression).
- Perform a complete physical examination.
- Record blood pressure (BP), heart rate (HR), respiratory rate (RR), and temperature

measurements.

- Record weight.
- Assess Eastern Cooperative Oncology Group (ECOG) Performance Status score (Appendix B Eastern Cooperative Oncology Group Performance Status Scale) and Karnofsky Performance Status score.
- Perform ECG to assess for cardiac arrhythmias or evidence of recent cardiac events.
- Draw blood samples for hematology, chemistry, and coagulation.
- Perform tumor assessment with MRI of the brain.
- Record recent medication history, including vitamins, herbal preparations, blood products, and other over the counter (OTC) drugs.
- Record all Adverse Events.

In accordance with good medical practice, any ongoing study drug-related AE present at End of Study or Early Study termination, including a clinically significant laboratory test abnormality, will be followed until resolved or until the event stabilizes and the overall clinical outcome has been ascertained.

#### 6.4.2. Survival Follow-up

Following the End of Study visit or Early Termination Visit, the subject will continue to be monitored by their physician to standard of care. The subject, a family member, and/or the PI will be contacted for survival information every 3 months until three years from first dose. Anti-tumor therapy (description and dates) since the last contact will be collected at each survival follow up.

### 6.5. Safety Procedures

#### 6.5.1. Physical Examination

A complete physical examination will be performed at screening and at study termination or early study termination and results will be recorded by the investigator (or designee). The results of the physical examinations will be used for safety monitoring purposes only. At each study visit, according to good medical practice, the subject's general health (e.g., appearance, adequacy of hydration, presence of illness or injury, temperature, vital signs indicative of a concurrent illness, etc.) will be assessed.

#### 6.5.2. Vital Signs

Blood pressure and HR measurements should be obtained with the subject's arm unconstrained by clothing or other material. The measurements will be obtained with the appropriate cuff size from the opposite arm from that used for blood sampling, where possible, which is supported at the level of the heart.

#### 6.5.3. Disease Assessment

Patients will be assessed with MRI per the modified RANO criteria as detailed in Appendix C.

Objective response rate (ORR) is defined as the proportion of patients with a CR or PR. The primary analysis for response will determine the point estimate and 95% confidence interval (CI) for the response rate. Progression free survival (PFS) is defined as the time from dosing of $^{186}$RNL to documented disease progression as determined by the investigator, clear clinical progression in the absence of a brain MRI determination of progression, or death from any cause, whichever occurs first.

If possible, patients who have a clinical determination of progression should undergo an MRI assessment to correlate radiographic findings with clinical findings. If a clinical determination of progression from a patient is confirmed by MRI, the date of MRI will be considered as the progression date.

Data for patients who are still alive and free from disease progression at the time of data cutoff date will be administratively censored on last assessment. Data for patients who are lost to follow-up prior to documented disease progression will be censored at the last disease assessment date when the patient is known to be disease progression-free.

# 7. MANAGEMENT OF INTERCURRENT EVENTS

Comprehensive assessments of any apparent toxicity experienced by the subject will be performed throughout the course of the study. Study site personnel will report any clinical Adverse Events, whether observed by the investigator or reported by the subject.

## 7.1. Grading of Toxicity

Clinical AEs or abnormal laboratory test results will be assessed by the principal investigator or other designated physician, in accordance with the current CTCAE version (currently v4.0) criteria.

## 7.2. Monitoring and Treatment of Toxicity

A physician (either the investigator or a physician designated by the investigator) will manage and treat any toxicity as clinically indicated.

## 7.3. Adverse Events

A physician (either the investigator or physician designated by the investigator) will assess the seriousness, severity, and causality of an AE based on the following definitions.

### 7.3.1. Defining Adverse Events

An adverse event (AE) is any undesirable event occurring to or in a subject enrolled in a clinical trial, whether or not the event is considered related to the $^{186}$RNL. This includes the time periods beginning after the first administration of study drug until resolution of all study related AEs.

Adverse events include the following types of occurrences:

- Suspected adverse reactions
- Other medical experiences, regardless of their relationship to the study drug, such as injury, causes for surgery, accidents, increased severity of pre-existing symptoms, apparently unrelated illnesses, and significant abnormalities in clinical laboratory values, physiological testing, or physical examination findings
- Reactions from drug overdose, abuse, withdrawal, sensitivity, or toxicity

#### 7.3.1.1. Serious Adverse Events

A serious adverse event (SAE) is any adverse experience that occurs at any dose and results in any of the following outcomes:

- Death: This includes any death that occurs during the conduct of the clinical study, including deaths that appear to be completely unrelated to the study drug (e.g., car accident). However, deaths that occur due to disease progression are not considered SAEs but will be reported as a death on study. If a subject dies during the study, and an autopsy is performed, the autopsy results will be sent to the PI. Possible evidence of organ toxicity and the potential relationship of the toxicity to the study drug are of particular interest. The autopsy report should distinguish between the relationship between the underlying diseases, their side effects, and the cause of death.
- Life-threatening adverse experience: This includes any AE during which the subject is, in the view of the PI, at immediate risk of death from the event as it occurs. This definition does not include any event that may have caused death if it had occurred in a more severe form.
- Persistent or significant disability or incapacity.
- Inpatient hospitalization or prolongation of existing hospitalization.
- Congenital anomaly or birth defect.
- Other medically important event which, according to appropriate medical judgment, may require medical or surgical intervention to prevent one of the outcomes listed above.
- Pregnancy occurring in subjects treated with $^{186}$RNL should be reported using the serious adverse event reporting form.

All SAEs will be reported to Plus Therapeutics within 24 hours by the site PI, study coordinator, or their designee by contacting:

Norman LaFrance, MD, ME, FACP, FACNP, FACNM
Plus Therapeutics, Chief Medical Officer, SVP
(215) 808-0955
safety_inbox@plustherapeutics.com

#### 7.3.1.2. Nonserious Adverse Events

A nonserious AE includes any AE that is not defined as an SAE.

#### 7.3.1.3. Unexpected Adverse Events

An unexpected AE is any AE that is not identified in nature, severity, or frequency.

### 7.3.2. Documenting All Adverse Events

All AEs will be recorded as descriptive findings (e.g., symptoms, laboratory, physical exam, vitals abnormalities) or diagnoses if etiology is known. Included are all AEs that occur after the start of treatment and through the End of Study visit for the patient. All AEs of any severity and AEs that are assessed as serious or not serious will be recorded.

Note: Unchanged, chronic conditions and cancer symptoms present at baseline are NOT AEs and will not be recorded unless there is an exacerbation or worsening in severity of a chronic condition or cancer symptom after the first administration of study drug until 30 days after the last dose of study drug. Chronic conditions and/or cancer symptoms that exacerbate or worsen in severity will be documented as a "worsening" condition. Death due to disease progression and measures of disease progression collected as efficacy endpoints (e.g., increasing tumor size or new lesions) are not considered adverse events, but will be collected as termination reasons (if applicable) and/or noted in tumor assessment appropriate. Other reasons for death occurring during the AE reporting period are SAEs and will be reported as such.

#### 7.3.2.1. Grading of Adverse Events

Severity of AEs or clinically significant laboratory test results will be assessed in accordance with the grading scale presented in the current CTCAE version (currently v4.0). Clinically significant abnormal laboratory results and lab results requiring an intervention will be recorded as AEs and should describe whether the lab result was increased or decreased. The following definitions for rating severity of AEs will be used for events not covered in the CTCAE.

- Grade 1: Mild; awareness of signs or symptoms that are easily tolerated, are of minor irritant type, cause no loss of time from usual activities, do not require medication or further medical evaluation, and/or are transient.
- Grade 2: Moderate; signs or symptoms sufficient to interfere with function but not activities of daily living.
- Grade 3: Severe; signs or symptoms sufficient to interfere with activities of daily living; signs and symptoms may be of a systemic nature or require further medical evaluation and/or treatment.
- Grade 4: Disabling or with life-threatening consequences. (This definition does not include any event that might have caused death if it had occurred in a more severe form.)
- Grade 5: Death

#### 7.3.2.2. Relationship to Study Treatment

Using the following criteria, investigators will assess whether there is a reasonable possibility that the study drug ($^{186}$RNL) caused or contributed to the AE.

YES

The time sequence between the onset of the AE and study drug administration is consistent with the event being related to study drug; and/or there is a possible biologic mechanism for study drug causing or contributing to the AE; and the AE may or may not be attributed to concurrent/underlying illness, other drugs, or procedures.

NO

Another cause of the AE is most likely; and/or the time sequence between the onset of the AE and study drug administration is inconsistent with a causal relationship; and/or a causal relationship is considered biologically unlikely.

#### 7.3.2.3. Abnormal Laboratory Test Results as Adverse Events

The PI will monitor the laboratory test results and determine the clinical significance of any result that falls outside of the reference range. In accordance with good medical practice, any clinically significant abnormal laboratory test results will be followed until resolved or stabilized. Abnormal laboratory test results will not be reported as AEs unless, in the opinion of the PI, the results constitute or are associated with a clinically relevant condition or require intervention.

In the event of unexplained, clinically significant abnormal laboratory test results, the tests will be repeated immediately and followed up until the values have returned to within the reference range or to baseline for that subject.

### 7.3.3. Reporting and Documenting Serious Adverse Events

Serious adverse events (SAE) that occur at any time point after the first dose of study drug until 30 days after the last dose of study drug will be reported. SAEs will be reported as per institutional policy and as required under the Data Safety Monitoring Plan (Section 10.6 for DSMP).

All known subject information (listed below) will be submitted within 24 hours of knowledge of the SAE occurrence. The following information will also be recorded (or as much as possible to obtain and still report the event within 24 hours):

- Subject’s Demographic Data
- Subject's weight
- Description of SAE, including date of onset and duration, severity, and outcome
- All dosing data of study drugs administered up to the date the SAE occurred
- Action taken regarding study drug administration
- Relationship of SAE to study drugs
- Concomitant medications, including regimen and indication
- Intervention, including concomitant medications used to treat SAE
- Pertinent laboratory data diagnostic tests conducted and date
- Pertinent medical history of subject
- Date of hospital admission discharge (if applicable)

- Date of death (if applicable)

Additionally, the following steps will be taken at the PI's discretion:

- Appropriate diagnostic tests and therapeutic measures.
- Appropriate consultation and follow-up evaluations until the events are resolved, stabilized, or otherwise explained by the principal investigator.
- SAE report review and evaluation of the relationship of the SAE to study treatment and to the underlying disease.
- All SAEs and unexpected problems reported promptly the PI's IRB/IEC, as appropriate (ICH Guidelines, Good Clinical Practice).

Other actions regarding SAEs might include the following:

- Protocol amendment
- Discontinuation or suspension of the protocol
- Modification of informed consent to include recent findings
- Informing current study participants of new findings
- Identification of specific AEs as drug-related

#### 7.3.4. Follow Up of Adverse Events

All AEs will be followed until they are resolved or determined to be irreversible or otherwise explained by the principal investigator.

### 7.4. Concomitant and excluded therapy

All medications and blood products (prescription and over the counter including herbal preparations) taken within 14 days of treatment will be recorded by the investigator (or designee). The reason(s) for treatment, dosage, and dates of treatment will be recorded in the source documents. In addition, concomitant medications used to treat adverse events occurring up to 30 days after the last dose of study drug will be recorded.

## 8. REMOVING SUBJECTS FROM THE STUDY

### 8.1. Criteria for termination

Subjects are free to discontinue (withdraw) at any time during this clinical trial. If a subject withdraws from participation in the study during the treatment period, he or she will be encouraged to return for an Early Termination visit for evaluation of safety (Section 6.4 End of Study, Early Study Termination, and Survival Follow-up).

The investigator has the right to discontinue any subject from study drug administration or study participation. Reasons for subject discontinuation may include, but are not limited to, the following:

- Clinically significant deterioration of the subject's condition
- Disease progression
- Requirement for other anti-tumor therapy during the study
- Noncompliance
- Pregnancy
- Significant AE
- Subject's right to withdraw from the study at any time, with or without stated reason
- Significant protocol violation
- Lost to follow-up
- Death
- Any other reason that, in the opinion of the PI, would justify the removal of a subject from the study. The primary consideration in any determination to discontinue a subject's participation must be the health and welfare of the subject.

All subjects will be instructed on the importance of complying with the requirements of the study. It is expected that subjects will complete all the necessary visits. If a subject does not return for follow-up visits as directed or does not adhere to the study requirements, the PI will determine if early withdrawal should occur.

### 8.2. Documentation

The primary reason for early removal of a subject from the study will be documented clearly and will be completed for any subject who has received any amount of drug during the treatment period. If the reason for early withdrawal is an AE or an abnormal laboratory value, the specific event or test result will also be recorded.

### 8.3. Procedures for subjects who withdraw early

Following early termination, the subject will be informed about which evaluations are necessary to monitor his or her safety. In addition, subjects will be encouraged to complete any procedures or evaluations outlined in Section 6.4, End of Study, Early Study Termination, and Survival Follow-up.

### 8.4. Replacement of subjects

Subjects who have not received $^{186}$RNL will be replaced. Subject enrollment numbers are unique and will not be re-assigned.

## 9. CONDITIONS FOR INITIATING, MODIFYING, OR TERMINATING THE STUDY

### 9.1. Institution Review

The PI will submit this protocol, any protocol modifications, and the subject Informed Consent form to be used in this study to the central Institutional Review Board (cIRB) for review and approval. If necessary, per local IRB guidelines, once the cIRB review is complete a submission to the local IRB will follow. A letter confirming IRB approval of the protocol and an IRB

approved-Informed Consent form will remain on file with the site.

### 9.2. Informed Consent

The investigator or his or her designee will explain to the subject, in the presence of a witness, the purpose and nature of the study, the study procedures, the possible adverse effects, and all other elements of consent as defined in 21 CFR Part 50 and Clinical Trial Directive or ICH guidelines before enrolling that subject in the study. It is the investigator's (or designee's) responsibility to obtain informed written consent from each subject, or if appropriate, the subject's parent or legal guardian.

### 9.3. Modifications

Any changes to the protocol will be made in accordance with FDA regulations concerning Phase 2 studies.

### 9.4. Deviations

The PI will consider any planned deviations from the protocol on a case-by-case basis. If a planned deviation is allowed by the PI, the rationale for such deviation will be thoroughly documented in the clinical notes. For unplanned deviations or violations, the investigator or other health professional in attendance will contact the local DSMC and IRB as soon as possible to discuss the associated circumstances. The PI and the DSMC will then decide whether the subject should continue to participate in the study. All protocol deviations and the reasons for such deviations will be noted in the source documents.

### 9.5. Termination

Although there are no predefined criteria for termination, if the investigator(s) discover conditions during the study that indicate it should be discontinued, an appropriate procedure for terminating the study will be instituted, including notification of the appropriate regulatory agencies and IRB.

## 10. INVESTIGATOR'S RESPONSIBILITIES

### 10.1. Responsibilities/Performance

The PI will ensure that this study is conducted in accordance with all regulations governing the protection of human subjects. The investigator will adhere to the basic principles of "Good Clinical Practice," as outlined in Title 21 of the Code of Federal Regulations (CFR), Part 312, Subpart D, "Responsibilities of Sponsors and Investigators"; 2 1 CFR, Part 50, "Protection of Human Subjects"; 21 CFR, Part 56, "Institutional Review Boards"; and the US Food and Drug Administration (FDA) guideline entitled "Good Clinical Practice: Consolidated Guideline". Additionally, this study will be conducted in compliance with the Declaration of Helsinki and with any local laws and regulations of the country in which the research is conducted. The PI will ensure that all work and services described in or associated with this protocol will be conducted in

accordance with the investigational plan, applicable regulations, and the highest standards of medical and clinical research practice. The PI is responsible for the control of drugs under investigation. The investigator will provide copies of the study protocol and Investigator's Brochure to all staff responsible for study conduct.

### 10.2. Confidentiality

The PI will ensure that each subject's anonymity will be maintained, and each subject's identity will be protected from unauthorized parties. A number will be assigned to each subject upon study entry and the number and the subject's initials will be used to identify the subject for the duration of the study. Documents (e.g., signed consent form) will be maintained by the investigator in strict confidence.

### 10.3. Institutional Review

The investigator will submit this protocol, any protocol modifications, and any accompanying material provided to the subject (e.g., informed consent form, subject information sheets, descriptions of the study used to obtain informed consent) to the appropriate central IRB for review and approval. A letter confirming IRB approval of the protocol and subject consent forms, and an IRB approved informed consent form will be provided to the appropriate review departments prior to the enrollment of subjects into the study.

### 10.4. Informed Consent and Permission to Use Protected Health Information

It is the responsibility of the PI to obtain written informed consent from each subject participating in this study after adequate explanation, in lay language, of the methods, objectives, anticipated benefits, and potential hazards of the study. The investigator will also explain that the subject is completely free to refuse to enter the study or to discontinue participation at any time (for any reason) and receive alternative conventional therapy as indicated. Prior to study participation, each subject will sign an IRB-approved informed consent form, which will be in form and substance acceptable to the IRB. For subjects not qualified or able to give legal consent, consent must be obtained from a parent, legal guardian, or custodian.

The PI or designee will explain to the subject before enrollment into the study that for evaluation of study results, the subject's protected health information obtained during the study may be shared with regulatory agencies, and IECs/IRBs. It is the investigator's (or designee's) responsibility to obtain permission to use protected health information per HIPAA from each subject, or if appropriate, the subjects' parent or legal guardian

### 10.5. Source Documentation and Investigator Files

The investigator will maintain adequate and accurate records to fully document the conduct of the study and to ensure that study data can be subsequently verified. These documents will be classified into two separate categories: (1) investigator study file and (2) subject clinical source documents that corroborate data collected.

Subject clinical source documents may include hospital clinic patient records; physician's and nurse's notes; appointment book; original laboratory, ECG, EEG, radiology, pathology, and special assessment reports; pharmacy dispensing records; subject diaries; signed informed consent forms; consultant letters; and subject screening, and enrollment logs.

The following will be documented in source documents at the site:

- Medical history/physical condition and diagnosis of the subject before involvement in the study sufficient to verify protocol entry criteria (if not already present)
- Study number, assigned subject number, and verification that written informed consent was obtained (each recorded in dated and signed notes on the day of entry into the study)
- Progress notes for each subject visit (each dated and signed)
- Review of laboratory test results
- Adverse events (action taken and resolution)
- Concomitant medications (including start and stop dates)
- Condition of subject upon completion of or early termination from the study

### 10.5.1. Subject Exclusion Log

The investigator or designee will keep a record listing all patients considered for entry into the study but subsequently excluded. The reason for each exclusion will be recorded in the Subject Exclusion Log.

## 10.6. Data Safety Monitoring

The Data and Safety Monitoring Board (DSMB) is an independent group of experts that advises the PI and the study investigators. The members of the DSMB serve in an individual capacity and provide their expertise and recommendations. The primary responsibilities of the DSMB are to 1) periodically review and evaluate the accumulated study data for participant safety, study conduct and progress, and, when appropriate, efficacy, and 2) make recommendations to the PI concerning the continuation, modification, or termination of the trial. The DSMB considers study-specific data as well as relevant background knowledge about the disease, test agent, or patient population under study. The DSMB is responsible for defining its deliberative processes, including event triggers that would call for an unscheduled review, stopping guidelines, unmasking (unblinding), and voting procedures prior to initiating any data review. The DSMB is also responsible for maintaining the confidentiality of its internal discussions and activities as well as the contents of reports provided to it. During the trial, the DSMB will review cumulative study data to evaluate safety, study conduct, and scientific validity and integrity of the trial. As part of this responsibility, DSMB members must be satisfied that the timeliness, completeness, and accuracy of the data submitted to them for review are sufficient for evaluation of the safety and welfare of study participants. The DSMB will also assess the performance of overall study operations and any other relevant issues, as necessary. Items reviewed by the DSMB include:

- Interim/cumulative data for evidence of study-related adverse events
- Interim/cumulative data for evidence of efficacy according to pre-established statistical guidelines, if appropriate

- Data quality, completeness, and timeliness
- Performance of individual centers
- Adherence to the protocol
- Factors that might affect the study outcome
- Factors external to the study such as scientific or therapeutic developments that may impact participant safety or the ethics of the study

At the conclusion of a DSMB meeting, the DSMB will discuss its findings and recommendations with PI and the study investigators. If the PI is not represented at the meeting, the DSMB Chair will contact the PI immediately after the meeting to debrief. The DSMB will issue a written summary report that identifies topics discussed by the DSMB and describes their individual findings, overall safety assessment and recommendations. The rationale for recommendations will be included when appropriate. This report will generally not include confidential information. The DSMB Chair or designee is responsible for drafting, circulating, and obtaining approval from other DSMB members within two (2) weeks of the meeting. The final summary report will be forwarded to a designated study team representative (usually the PI) and to other appropriate staff. The study team representative will be responsible for disseminating the DSMB summary report to site investigators who will, in turn, submit the report to their local IRBs. The sponsor or their designee will forward the summary report including routine and nominal findings to the Food and Drug Administration (FDA) and to any other collaborators.

The DSMB Chair will notify the study team monitoring the study and the PI of any findings of a serious and immediate nature or recommendations to discontinue all or part of the trial. The study team will immediately inform site staff. In addition to verbal communications, recommendations to discontinue or substantially modify the design or conduct of a study must be conveyed to the PI in writing by e-mail, fax, or courier on the day of the DSMB meeting.

## 11. REFERENCES

Bobo RH, Laske DW, Akbasak A, Morrison PF, Dedrick RL, Oldfield EH. 1994. Convection-enhanced delivery of macromolecules in the brain. Proc Natl Acad Sci. 91(6):2076–2080. doi:10.1073/pnas.91.6.2076.

Chang JE, Khuntia D, Robins HI, Mehta MP. 2007. Radiotherapy and radiosensitizers in the treatment of glioblastoma multiforme. Clin Adv Hematol Oncol HO. 5(11):894–902, 907–915.

Chopra A. 2004. 188Re-Labeled humanized monoclonal anti-epidermal growth factor receptor antibody. In: Molecular Imaging and Contrast Agent Database (MICAD). Bethesda (MD): National Center for Biotechnology Information (US). [accessed 2022 Sep 16]. http://www.ncbi.nlm.nih.gov/books/NBK23376/.

Cloughesy TF, Brenner A, de Groot JF, Butowski NA, Zach L, Campian JL, Ellingson BM, Freedman LS, Cohen YC, Lowenton-Spier N, et al. 2020. A randomized controlled phase III study of VB-111 combined with bevacizumab vs bevacizumab monotherapy in patients with recurrent glioblastoma (GLOBE). Neuro-Oncol. 22(5):705–717. doi:10.1093/neuonc/noz232.

Colnat-Coulbois S, Klein O, Braun M, Thouvenot P, Marchal J-C. 2010. Management of Intramedullary Cystic Pilocytic Astrocytoma With Rhenium-186 Intracavitary Irradiation: Case Report. Neurosurgery. 66(5):E1023–E1024. doi:10.1227/01.NEU.0000367800.59074.C2.

Colnot DR, Quak JJ, Roos JC, van Lingen A, Wilhelm AJ, van Kamp GJ, Huijgens PC, Snow GB, van Dongen GA. 2000. Phase I therapy study of 186Re-labeled chimeric monoclonal antibody U36 in patients with squamous cell carcinoma of the head and neck. J Nucl Med Off Publ Soc Nucl Med. 41(12):1999–2010.

Friedman HS, Prados MD, Wen PY, Mikkelsen T, Schiff D, Abrey LE, Yung WKA, Paleologos N, Nicholas MK, Jensen R, et al. 2009. Bevacizumab Alone and in Combination With Irinotecan in Recurrent Glioblastoma. J Clin Oncol. 27(28):4733–4740. doi:10.1200/JCO.2008.19.8721.

Hou LC, Veeravagu A, Hsu AR, Tse VCK. 2006. Recurrent glioblastoma multiforme: a review of natural history and management options. Neurosurg Focus. 20(4):E3. doi:10.3171/foc.2006.20.4.2.

I. Mehta A, D. Choi B, Ajay D, Raghavan R, Brady M, H. Friedman A, Pastan I, D. Bigner D, H. Sampson J. 2012. Convection Enhanced Delivery of Macromolecules for Brain Tumors. Curr Drug Discov Technol. 9(4):305–310. doi:10.2174/157016312803305951.

James CD. 2012. Nanoparticles for treating brain tumors: unlimited possibilities. Neuro-Oncol. 14(4):389–389. doi:10.1093/neuonc/nos075.

Kang S-H, Ahn CW. 2002. An Investigation of the Traditional Algorithm-Based Designs for Phase 1 Cancer Clinical Trials. Drug Inf J. 36(4):865–873. doi:10.1177/009286150203600415.

Lam MGEH, de Klerk JMH, van Rijk PP. 2004. 186Re-HEDP for metastatic bone pain in breast cancer patients. Eur J Nucl Med Mol Imaging. 31(S1):S162–S170. doi:10.1007/s00259-004-1539-

4.

Liu P, Chen G, Zhang J. 2022. A Review of Liposomes as a Drug Delivery System: Current Status of Approved Products, Regulatory Environments, and Future Perspectives. Molecules. 27(4):1372. doi:10.3390/molecules27041372.

Louis DN, Perry A, Wesseling P, Brat DJ, Cree IA, Figarella-Branger D, Hawkins C, Ng HK, Pfister SM, Reifenberger G, et al. 2021. The 2021 WHO Classification of Tumors of the Central Nervous System: a summary. Neuro-Oncol. 23(8):1231–1251. doi:10.1093/neuonc/noab106.

NCI. 2022. Cancer Stat Facts: Brain and Other Nervous System Cancer. Natl Cancer Inst. https://seer.cancer.gov/statfacts/html/brain.html.

Ostrom QT, Patil N, Cioffi G, Waite K, Kruchko C, Barnholtz-Sloan JS. 2020. CBTRUS Statistical Report: Primary Brain and Other Central Nervous System Tumors Diagnosed in the United States in 2013–2017. Neuro-Oncol. 22(Supplement_1):iv1–iv96. doi:10.1093/neuonc/noaa200.

Phillips WT, Goins B, Bao A, Vargas D, Guttierez JE, Trevino A, Miller JR, Henry J, Zuniga R, Vecil G, et al. 2012. Rhenium-186 liposomes as convection-enhanced nanoparticle brachytherapy for treatment of glioblastoma. Neuro-Oncol. 14(4):416–425. doi:10.1093/neuonc/nos060.

Proust F, Coche-Dequeant B, Carpentier P, Laquerrière A, Derlon JM, Blond S, Christiaens J, Fréger P. 1998. [Combination treatment for pilocytic astrocytoma: stereotaxic radiosurgery and endocavitary radiotherapy]. Neurochirurgie. 44(1):50–54.

Quant EC, Norden AD, Drappatz J, Muzikansky A, Doherty L, LaFrankie D, Ciampa A, Kesari S, Wen PY. 2009. Role of a second chemotherapy in recurrent malignant glioma patients who progress on bevacizumab. Neuro-Oncol. 11(5):550–555. doi:10.1215/15228517-2009-006.

Sgouros G, Bodei L, McDevitt MR, Nedrow JR. 2020. Radiopharmaceutical therapy in cancer: clinical advances and challenges. Nat Rev Drug Discov. 19(9):589–608. doi:10.1038/s41573-020-0073-9.

Shaw E, Scott C, Souhami L, Dinapoli R, Kline R, Loeffler J, Farnan N. 2000. Single dose radiosurgical treatment of recurrent previously irradiated primary brain tumors and brain metastases: final report of RTOG protocol 90-05. Int J Radiat Oncol. 47(2):291–298. doi:10.1016/S0360-3016(99)00507-6.

Shi L, Zhang Z, Zhuang D, Cheng H, Gao Y, Wu M. 2002. [Radiotherapy with (186)Re-lipiodol II for primary liver cancer patients]. Zhonghua Wai Ke Za Zhi. 40(11):814–816.

Strosberg J, El-Haddad G, Wolin E, Hendifar A, Yao J, Chasen B, Mittra E, Kunz PL, Kulke MH, Jacene H, et al. 2017. Phase 3 Trial of $^{177}$Lu-Dotatate for Midgut Neuroendocrine Tumors. N Engl J Med. 376(2):125–135. doi:10.1056/NEJMoa1607427.

Stupp R, Taillibert S, Kanner AA, Kesari S, Steinberg DM, Toms SA, Taylor LP, Lieberman F,

Silvani A, Fink KL, et al. 2015. Maintenance Therapy With Tumor-Treating Fields Plus Temozolomide vs Temozolomide Alone for Glioblastoma: A Randomized Clinical Trial. JAMA. 314(23):2535. doi:10.1001/jama.2015.16669.

Stupp R, Weller M, Belanger K, Bogdahn U, Ludwin SK, Lacombe D, Mirimanoff RO. 2005. Radiotherapy plus Concomitant and Adjuvant Temozolomide for Glioblastoma. N Engl J Med.:10.

Vogelbaum MA, Iannotti CA. 2012. Convection-enhanced delivery of therapeutic agents into the brain. In: Handbook of Clinical Neurology. Vol. 104. Elsevier. p. 355–362. [accessed 2022 Sep 15]. https://linkinghub.elsevier.com/retrieve/pii/B9780444521385000232.

Werner-Wasik M, Scott CB, Nelson DF, Gaspar LE, Murray KJ, Fischbach JA, Nelson JS, Weinstein AS, Curran WJ. 1996. Final report of a phase I/II trial of hyperfractionated and accelerated hyperfractionated radiation therapy with carmustine for adults with supratentorial malignant gliomas: Radiation therapy oncology group study 83-02. Cancer. 77(8):1535–1543. doi:10.1002/(SICI)1097-0142(19960415)77:8<1535::AID-CNCR17>3.0.CO;2-0.

**Appendix A**
SCHEDULE OF EVENTS

(SEE SEPARATE DOCUMENT)

## Appendix B

EASTERN COOPERATIVE ONCOLOGY GROUP (ECOG) PERFORMANCE SCORE SCALE

| Grade | Description |
|---|---|
| 0 | Fully active, able to carry on all pre-disease performance without restriction. |
| 1 | Restricted in physically strenuous activity but ambulatory and able to carry out work of a light or sedentary nature, e.g., light housework, office work. |
| 2 | Ambulatory and capable of all self-care but unable to carry out any work activities. Up and about more than 50% of waking hours. |
| 3 | Capable of only limited self-care, confined to bed or chair more than 50% of waking hours. |
| 4 | Completely disabled. Cannot carry on any self-care. Totally confined to bed or chair. |
| 5 | Dead |

# Appendix C
RANO TUMOR RESPONSE

**Table: Summary of the RANO Response Criteria**

| | CR | PR | SD | PD# |
|---|---|---|---|---|
| **T1-Gd +** | None | ≥50% decrease | <50% decrease-<25% increase | ≥25% increase* |
| **T2/FLAIR** | Stable or decrease | Stable or decrease | Stable or decrease | Increase* |
| **New Lesion** | None | None | None | Present* |
| **Corticosteroids** | None | Stable or decrease | Stable or decrease | **NA** |
| **Clinical Status** | Stable or increase | Stable or increase | Stable or increase | Decrease* |
| **Requirement for Response** | All | All | All | Any* |

CR=complete response; PR=partial response; SD=stable disease; PD=progressive disease

# Progression occurs when any of the criteria with * is present

NA: Increase in corticosteroids alone will not be taken into account in determining progression in the absence of persistent clinical deterioration

## Appendix D
PATIENT SAFETY SHEET

Precautions Following Discharge from Hospital After Radioactive Rhenium-Liposome Therapy

1. Avoid close contact with children and pregnant women for at least the first 3 days. An occasional hug is not harmful, but prolonged contact is discouraged.
2. Maintain a prudent distance from others for at least the first 3 days (e.g., two arm lengths or approximately six feet).
3. Sleep alone in a separate room for at least the first night. No sexual intercourse for the first 5 days.
4. Do not travel by airplane or mass transportation for at least the first 5 days.
5. Do not travel on a prolonged automobile trip (more than 2 hours at a time) with others for the first 5 days.
6. Have the sole use of a bathroom for at least the first 3 days. If this is not possible, clean the bathroom when finished. Any observed body fluids should be wiped up with a tissue and flushed down the toilet. Rinse the sink after use. Continue to maintain good bathroom hygiene for the first 3 days. Males should sit on the toilet to urinate.
7. Flush the toilet two times after each use for the first 3 days.
8. Good hydration (drinking plenty of liquids) is important and highly recommended. Frequent voiding of urine is helpful in eliminating the radioactivity from the bladder and the rest of the body.
9. At least one bowel movement a day is recommended to reduce colon radioactivity exposure. Laxatives may be necessary if you are constipated.
10. Use separate eating utensils during the first 3 days, or wash eating utensils and dishes in a dishwasher before others use them during this time.
11. Avoid mouth-to-mouth contact, i.e., kissing. Keep items that contact the mouth (e.g., toothbrush, drinking glasses) from use by other individuals for the first 5 days.
12. Clothing worn directly in contact with the skin during the first 5 days after the therapy dose should be laundered separate before reuse.

# Appendix E
## CATHETER AND INFUSION RATE DATA

**BrainLab SmartFlow™ Flex Ventricular Catheter, 0.021" x 7.5mm Tip (510K – K123605)**

**Dimensions and Priming Volume**

| Catalog Number | Outside Dia. | | Tip ID | | Total Length | | Distal Length | | Priming Volume |
|---|---|---|---|---|---|---|---|---|---|
| | (in.) | (mm) | (in) | (µm) | (in) | (mm) | (in) | (mm) | (ml) |
| 19772 | 0.082 | 2.08 | 0.021 | 536 | 23.0 | 584 | 10.0 | 254 | 0.131 |

**Diagram**

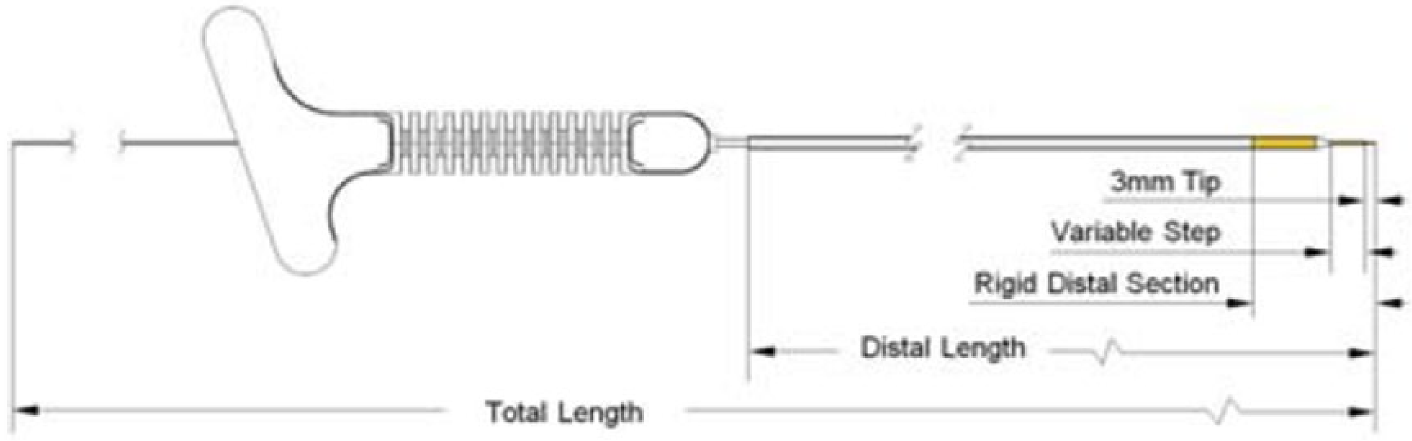


**Infusion Rates**

| Step | Rate per Catheter µL/min (mL/hour) | Interval (minutes) |
|---|---|---|
| 1 | 5 (0.30) | 20 |
| 2 | 10 (0.60) | 20 |
| 3 | 15 (0.90) | 20 |
| 4 | 20 (1.20) | Time to complete remainder of infusion volume |

**Catheter Label:**

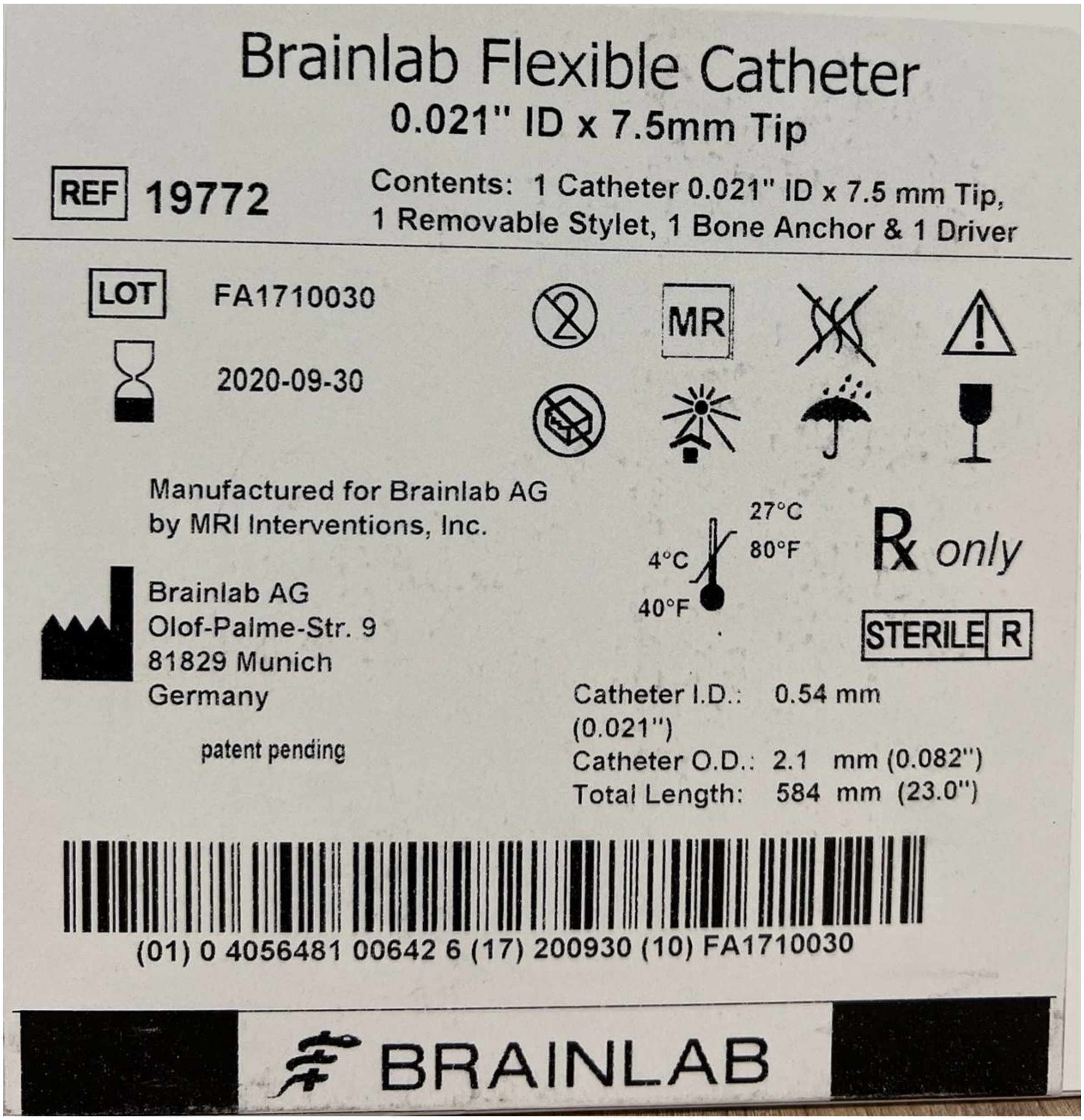

Supplement: Supplementary file 1 — Supplementary Information [file 41467_2025_57263_MOESM1_ESM.pdf]
