# Supplementary material for: Convection enhanced delivery of Rhenium (186Re) Obisbemeda (186RNL) in recurrent glioma: a multicenter, single arm, phase 1 clinical trial
Source: Nat Commun. 2025 Mar 7;16:2079. doi: 10.1038/s41467-025-57263-1 (PMC11889265; doi:10.1038/s41467-025-57263-1)
Supplement: Supplementary file 2 — Reporting Summary [file 41467_2025_57263_MOESM2_ESM.pdf]

nature portfolio

Corresponding author(s): Andrew J. Brenner

Last updated by author(s): Jan 16, 2025

Reporting Summary

Nature Portfolio wishes to improve the reproducibility of the work that we publish. This form provides structure for consistency and transparency in reporting. For further information on Nature Portfolio policies, see our Editorial Policies and the Editorial Policy Checklist.

Statistics

For all statistical analyses, confirm that the following items are present in the figure legend, table legend, main text, or Methods section.

n/a	Confirmed	
☐	☒	The exact sample size (*n*) for each experimental group/condition, given as a discrete number and unit of measurement
☐	☒	A statement on whether measurements were taken from distinct samples or whether the same sample was measured repeatedly
☐	☒	The statistical test(s) used AND whether they are one- or two-sided<br>*Only common tests should be described solely by name; describe more complex techniques in the Methods section.*
☐	☒	A description of all covariates tested
☐	☒	A description of any assumptions or corrections, such as tests of normality and adjustment for multiple comparisons
☐	☒	A full description of the statistical parameters including central tendency (e.g. means) or other basic estimates (e.g. regression coefficient) AND variation (e.g. standard deviation) or associated estimates of uncertainty (e.g. confidence intervals)
☐	☒	For null hypothesis testing, the test statistic (e.g. F, t, r) with confidence intervals, effect sizes, degrees of freedom and P value noted<br>*Give P values as exact values whenever suitable.*
☒	☐	For Bayesian analysis, information on the choice of priors and Markov chain Monte Carlo settings
☒	☐	For hierarchical and complex designs, identification of the appropriate level for tests and full reporting of outcomes
☒	☐	Estimates of effect sizes (e.g. Cohen's d, Pearson's r), indicating how they were calculated

Our web collection on statistics for biologists contains articles on many of the points above.

Software and code

Policy information about availability of computer code

Data collection	Study data were collected and managed using REDCap (Version 13.7.31, Nashville, TN) electronic data capture tools hosted at University of Texas Health Science Center at San Antonio1,2. REDCap (Research Electronic Data Capture) is a secure, web-based software platform designed to support data capture for research studies, providing 1) an intuitive interface for validated data capture; 2) audit trails for tracking data manipulation and export procedures; 3) automated export procedures for seamless data downloads to common statistical packages; and 4) procedures for data integration and interoperability with external sources.<br><br>References<br>1PA Harris, R Taylor, R Thielke, J Payne, N Gonzalez, JG. Conde, Research electronic data capture (REDCap) – A metadata-driven methodology and workflow process for providing translational research informatics support, J Biomed Inform. 2009 Apr;42(2):377-81.<br>2PA Harris, R Taylor, BL Minor, V Elliott, M Fernandez, L O'Neal, L McLeod, G Delacqua, F Delacqua, J Kirby, SN Duda, REDCap Consortium, The REDCap consortium: Building an international community of software partners, J Biomed Inform. 2019 May 9 [doi: 10.1016/j.jbi.2019.103208]
Data analysis	We used SAS (Version 9.4, SAS Institute, Cary, NC) for statistical analysis, graphics, and tabling. Data from REDCap were imported into SAS with the SAS import procedure. The SAS Output Delivery System and the report procedure were used to make publication quality tables in MS Word directly from SAS databases.

For manuscripts utilizing custom algorithms or software that are central to the research but not yet described in published literature, software must be made available to editors and reviewers. We strongly encourage code deposition in a community repository (e.g. GitHub). See the Nature Portfolio guidelines for submitting code & software for further information.

Data

Policy information about availability of data

All manuscripts must include a data availability statement. This statement should provide the following information, where applicable:

- Accession codes, unique identifiers, or web links for publicly available datasets
- A description of any restrictions on data availability
- For clinical datasets or third party data, please ensure that the statement adheres to our policy

All individual participant data that underlie the results reported, after de-identification, will be shared by the lead contact, Andrew J. Brenner, MD, PhD (BrennerA@uthscsa.edu) via an appropriate dataset repository, upon request.

Research involving human participants, their data, or biological material

Policy information about studies with human participants or human data. See also policy information about sex, gender (identity/presentation), and sexual orientation and race, ethnicity and racism.

Reporting on sex and gender	We do not use "sex" or "gender" as descriptors in the current manuscript. We assess gender by stating: "Among the 21 patients treated in cohorts 1 to 6,...66.7% were male...", as gender was self-reported to the PI. Sex and gender were not considered in the study design, which was open to any biological sex/gender. For overall numbers of gender (as self-reported): 7 female and 14 male. We did not analyze the data on a sex- and/or gender-basis; due to the inadequate numbers of a Phase 1, analysis was descriptive and included all patients.
Reporting on race, ethnicity, or other socially relevant groupings	For reporting on race, ethnicity, or other socially relevant groups, we collected the following demographic data: Race: White (19); Black (0); American Indian, Aleutian, or Eskimo (0); Hawaiian (0); Other Asian (1); Pacific Islander; Other; Unknown (1). Ethnicity: Non-Spanish (18); Spanish/Hispanic/Latino (2); Other (0); Unknown (1). No variables were used as proxies for other socially constructed/relevant variables. The terms in the form of questions were provided during the screening visit by the intake person directly to the participant and answered directly by the participant; categories were self-reported. We did not analyze the data on race or ethnicity basis; due to the inadequate numbers of a Phase 1, analysis was descriptive and included all patients.
Population characteristics	For reporting on population characteristics, we collected the following data: age at time of screening (dd/mm/yyyy); date of initial diagnosis (dd/mm/yyyy); primary tumor site; histologic diagnosis; stage at initial diagnosis; current stage at screening; metastatic disease presence/absence/site; gene expression (IDH and MGMT status); prior radiation therapy for current cancer; additional biopsies or surgery related to current cancer and procedure date (dd/mm/yyyy); prior systemic cancer-related therapies and start/end dates (dd/mm/yyyy). Although we reported on age, gene expression, and stage at screening, we did not further analyze the data on population characteristics; due to the inadequate numbers of a Phase 1, analysis was descriptive and included all patients.
Recruitment	Recruitment occurred directly at the sites by investigators as part of standard clinical practice. Patients were provided with information about the trial and given the opportunity to ask questions to the investigator about the trial and its risks/benefits compared to other treatment options, including other clinical trials (investigational drugs).
Ethics oversight	The study was performed in accordance with ethical principles that have their origin in the Declaration of Helsinki and are consistent with International Council for Harmonisation (ICH)/GCP and applicable regulatory requirements, and was conducted in accordance with applicable national, state, and local laws. Written informed consent was obtained from each subject prior to the subject entering the study or the performance of any study related procedures. During screening, candidates received a copy of the Informed Consent Form (ICF) that was approved by the Investigator's Investigational Review Board (IRB). Each patient was fully informed about the full nature of the study, possible benefits risks, and asked for permission to use protected health information (in accordance with the Health Insurance Portability and Accountability Act or HIPAA). Candidates read and signed the ICF in the presence of a member of the study team after all patient or family questions were answered. Refusal to sign informed consent and permission excluded an individual from the study.<br><br>Prior to advancement to the next Cohort, the Data and Safety Monitoring Board (DSMB) was consulted. The DSMB is an independent group of experts that advises the PI and the study investigators. The members of the DSMB serve in an individual capacity and provide their expertise and recommendations. The primary responsibilities of the DSMB are to 1) periodically review and evaluate the accumulated study data for participant safety, study conduct and progress, and, when appropriate, efficacy, and 2) make recommendations to the PI concerning the continuation, modification, or termination of the trial. The DSMB considers study-specific data as well as relevant background knowledge about the disease, test agent, or patient population under study. The DSMB is responsible for defining its deliberative processes, including event triggers that would call for an unscheduled review, stopping guidelines, and voting procedures prior to initiating any data review. The DSMB reviewed cumulative study data to evaluate safety, study conduct, and scientific validity and integrity of the trial. As part of this responsibility, DSMB members must be satisfied that the timeliness, completeness, and accuracy of the data submitted to them for review are sufficient for evaluation of the safety and welfare of study participants. The DSMB also assessed the performance of overall study operations and any other relevant issues, as necessary. At the conclusion of a DSMB meeting, the DSMB discussed its findings and recommendations with PI and the study investigators. The DSMB issued a written summary report that identified topics discussed by the DSMB and described their individual findings, overall safety assessment, and recommendations regarding proceeding to the next cohort as applicable.

Note that full information on the approval of the study protocol must also be provided in the manuscript.

Field-specific reporting

Please select the one below that is the best fit for your research. If you are not sure, read the appropriate sections before making your selection.

[x] Life sciences   [ ] Behavioural & social sciences   [ ] Ecological, evolutionary & environmental sciences

For a reference copy of the document with all sections, see nature.com/documents/nr-reporting-summary-flat.pdf

Life sciences study design

All studies must disclose on these points even when the disclosure is negative.

Sample size	We used a 3+3 design with the default n=3 patients per Cohort. The doses were specified following a modified Fibonacci sequence and the number of Cohorts were specified in the study protocol based subject matter knowledge. The sample size was specified in the protocol following the 3+3 methodology. Power calculations were not made and were not used.
Data exclusions	Inclusion and exclusion criteria were specified in the protocol and followed. Exclusions were not made during statistical analysis.
Replication	Replication was not attempted. Results were reviewed by subject matter experts for consistency and reasonableness.
Randomization	Subjects were considered for inclusion in sequence as seen in the clinic. Randomization was not used.
Blinding	The study was not blinded. The Principal Investigator, Sponsor, statistician, and co-investigators knew the Cohort status of each patient as presented in draft results and presentations at project meetings.

Reporting for specific materials, systems and methods

We require information from authors about some types of materials, experimental systems and methods used in many studies. Here, indicate whether each material, system or method listed is relevant to your study. If you are not sure if a list item applies to your research, read the appropriate section before selecting a response.

Materials & experimental systems

n/a	Involved in the study	
[x]	[ ]	Antibodies
[x]	[ ]	Eukaryotic cell lines
[x]	[ ]	Palaeontology and archaeology
[x]	[ ]	Animals and other organisms
[ ]	[x]	Clinical data
[x]	[ ]	Dual use research of concern
[x]	[ ]	Plants

Methods

n/a	Involved in the study	
[x]	[ ]	ChIP-seq
[x]	[ ]	Flow cytometry
[ ]	[x]	MRI-based neuroimaging

Clinical data

Policy information about clinical studies

All manuscripts should comply with the ICMJEguidelines for publication of clinical research and a completedCONSORT checklist must be included with all submissions.

Clinical trial registration

NCT0190638

Study protocol

The full study protocol is not currently publicly available. The protocol will be shared by the lead contact, Andrew J. Brenner, MD, PhD (BrennerA@uthscsa.edu), upon request.

Data collection

Patients were enrolled in the study between March 5, 2015 and April 22, 2021 at two study sites, UT Heath San Antonio and UT Southwestern Medical Center. Recruitment occurred during these time periods and data collection followed the schedule of events as outlined in the protocol.

Outcomes

The primary objective was to determine the maximum tolerated dose of 186RNL by convection enhanced delivery (CED) at the time of planned stereotactic biopsy, when necessary, as standard of care. To assess maximum tolerated dose, we used standard safety measures of dose limiting toxicities.

Escalation was performed by increases in 186RNL activity (mCi), with the first three escalations achieved by dose doubling followed by a modified Fibonacci dose escalation scheme (i.e., dose increases of 67%, 50%, 40% and 33%). Dose escalation followed a standard 3+3 design, with escalation between Cohorts dependent on the number of observed dose limiting toxicities (DLTs):

For this study, a DLT is defined as:

• Grade 3 or greater acute CNS toxicity attributable to the study intervention which persists for 96 hours or more (see below discussion of delayed events) OR
• Grade 3 or greater non-CNS toxicity which is attributable to the study intervention.

If a DLT is observed in 1 out of 3 patients at a given dose level, up to an additional 3 patients will be enrolled and treated at that dose level.

If 2 out of 3-6 patients at that dose level have DLTs, the dose will be decreased to the previous dose level and up to 3 additional patients will be enrolled at that dose level for a total of 6 patients.

When up to 3 additional patients are added to a given dose level, if 1 of 6 patients has a DLT then the dose will be increased to the next dose level.

Given the possibility for radiation effects outside of the traditional DLT window, the following consideration will also be given:

• The DLT evaluation period for CNS toxicity will include 90 days between Cohorts. This will not limit accrual within a Cohort, but across successive Cohorts. As an example, the first patient of Cohort 3 cannot be enrolled until the first patient of the Cohort 2 has completed 90 days of CNS toxicity evaluation.
• If a patient within a Cohort experiences CNS toxicity that would be defined as dose limiting, the entire Cohort must complete 90 days evaluation before the successive Cohort may commence.

Secondary objectives included: (1) To assess the safety of single dose 186RNL by CED; (2) To assess the dose distribution of 186RNL by CED; (3) To determine the overall response rate by Radiographic Assessment in Neuro-Oncology (RANO) criteria following 186RNL treatment, (4) To determine disease specific progression-free survival after 186RNL treatment.

For disease assessment, study subjects were routinely assessed by MRI (standard of care) until disease progression according to RANO criteria. There was an additional follow up for overall survival up to a period of 36 months (3 years). Radiographic response to study drug (186RNL) was determined in comparison to pretreatment baseline. The smallest tumor measurement was used for determination of progression. If subsequent imaging studies demonstrated that progression occurred, the date of progression was noted as the date of the scan at which this issue was first raised. All measurable and non-measurable lesions were assessed using the same techniques as at baseline on the same MRI scanner, or at minimum with the same magnet strength, to reduce difficulties in interpreting changes. Assessment of response by RANO criteria was used, recognizing the potential of pseudo-progression significantly complicating the use of the RANO criteria.

Objective response rate (ORR) is defined as the proportion of patients with a CR or PR. The primary analysis for response determined the point estimate and 95% confidence interval (CI) for the response rate. Progression free survival (PFS) was defined as the time from dosing of 186RNL to documented disease progression as determined by the investigator, clear clinical progression in the absence of a brain MRI determination of progression, or death from any cause, whichever occurs first. If possible, patients who had a clinical determination of progression underwent an MRI assessment to correlate radiographic findings with clinical findings. If a clinical determination of progression from a patient was confirmed by MRI, the date of the MRI was considered as the progression date. Data for patients who are still alive and free from disease progression at the time of data cutoff date were administratively censored on last assessment. Data for patients who are lost to follow-up prior to documented disease progression were censored at the last disease assessment date when the patient is known to be disease progression-free.

Plants

Seed stocks	N/A PER ABOVE
Novel plant genotypes	N/A PER ABOVE
Authentication	N/A PER ABOVE

Magnetic resonance imaging

Experimental design

Design type	We did not perform fMRI; not applicable for this study.
Design specifications	We did not perform fMRI; not applicable for this study.
Behavioral performance measures	We did not perform fMRI; not applicable for this study.

Acquisition

Imaging type(s)	Structural, diffusion, perfusion
Field strength	3T preferred

Sequence & imaging parameters

We perform two different MRIs for the study: (1) Pre-treatment MRI, before the patient is treated with 186RNL, and (2) post-treatment MRI, at regular time points after the patient has been treated with 186RNL until progression (as per protocol). We also ask that all imaging should be completed on the same machine at subsequent time points, if possible.

Pre-treatment MRI:

Contrast: Gadolinium-based contrast agent
Dose: 0.1 mmol/kg
Technique of administration: Dose is injected via intravenous injection
Patient Position: Head-in supine
Imaging Field: Brain and upper C-spine with inferior margin at the end of C-2 spine
Acquisition Protocol:
1. 3 plane localizer
2. Axial 3D T1 PRE MPRAGE (TR 2100 ms, TE 5 ms, TI 1100 ms, Flip Angle: 15 degrees. Freq 256, Phase 256, Freq Dir A/P, FOV: 256 mm, Slice thickness: 1 mm)
3. Axial T2 FLAIR TSE (TR 10000 ms, TE 100 ms, TI 2500 ms, Flip Angle: 90/160 degrees, Freq 256, Phase 256, Freq Dir A/P, FOV: 240 mm, Slice thickness: 3 mm, Gap: 0)
4. Axial DWI EPI (TR >5000 ms, TE min. Freq 128, Phase 128, Freq Dir R/L, FOV: 240 mm, Slice thickness: 3 mm, Gap: 0)
5. DTI (Spin-echo EPI single shot, Any Matrix, Pixel size must be square, Slices = 3 mm or less, axial, cover entire brain, 6 or more Directions b=1000 + 1 b = 0, Repetition's allowed; scanning position supine and head-first only; no angulation; 16-bit DICOM only
6. PRELOAD 2 mL of gadolinium-based contrast agent at 3-5 cc/sec followed by 2 minutes delay
7. Axial DSC (Single-shot, gradient-echo, EPI); begin imaging and start injecting after 30 seconds, 5 mL contrast at 5 cc per second, followed by 30 mL saline flush. EPI (TR < 2000 ms, TE 30 ms, Flip Angle: 60 degrees. Freq 128, Phase 128, NEX = 1 with 120 Reps, Freq Dir A/P, FOV: 240 mm, Slice thickness: 5 mm, Gap: 0, Sufficient to cover tumor)
8. Axial 3D T1 MPRAGE post contrast (TR 2100 ms, TE 5 ms, TI 1100 ms, Flip Angle: 15 degrees. Freq 256, Phase 256, Freq Dir A/P, FOV: 256 mm, Slice thickness: 1 mm)
9. Axial T2 TSE (TR >3500 ms, TE 100 ms, Flip Angle: 90/160 degrees, Freq 256, Phase 256, Freq Dir A/P, FOV: 240 cm, Slice thickness: 3mm, Gap: 0)
10. Return patient from 60-90 minutes post contrast injection for delayed image; repeat Axial 3D T1 MPRAGE as above.

Post-treatment MRI:

Contrast: Gadolinium-based contrast agent
Dose: 0.1 mmol/kg
Technique of administration: Dose is injected via intravenous injection
Patient Position: Head-in supine
Imaging Field: Brain and upper C-spine with inferior margin at the end of C-2 spine

Acquisition Protocol:
1. 3 plane localizer
2. Axial 3D T1 PRE MPRAGE (TR 2100 ms, TE 5 ms, TI 1100 ms, Flip Angle: 15 degrees. Freq 256, Phase 256, Freq Dir A/P, FOV: 256 mm, Slice thickness: 1 mm)
3. Axial T2 FLAIR TSE (TR 10000 ms, TE 100 ms, TI 2500 ms, Flip Angle: 90/160 degrees. Freq 256, Phase 256, Freq Dir A/P, FOV: 240 mm, Slice thickness: 3 mm, Gap: 0)
4. Axial DWI EPI (TR >5000 ms, TE min. Freq 128, Phase 128, Freq Dir R/L, FOV 240 mm, Slice thickness: 3 mm, Gap: 0)
5. PRELOAD 2 mL of gadolinium contrast agent at 3-5 cc/sec followed by 2 minutes delay
6. Axial DSC (Single-shot, gradient-echo,EPI); begin imaging and start injecting after 30 seconds, 5 mL contrast at 5 cc per second, followed by 30 mL saline flush. EPI (TR <2000 ms, TE 30 ms, Flip Angle: 60 degrees. Freq 128, Phase 128, NEX=1 with 120 Reps, Freq Dir A/P, FOV: 240 mm, Slice thickness: 5 mm, Gap: 0, Sufficient to cover tumor)
7. Axial 3D T1MPRAGE post contrast (TR 2100 ms, TE 5 ms, TI 1100 ms, Flip Angle:15 degrees. Freq 256, Phase 256, Freq Dir A/P, FOV: 256 mm, Slice thickness: 1 mm
8. Axial T2 TSE (TR >3500 ms, TE 100 ms, Flip Angle: 90/160 degrees. Freq 256, Phase 256, Freq Dir A/P, FOV: 240 mm, Slice thickness: 3 mm, Gap: 0)
9. Return patient from 60-90 minutes post contrast injection for delayed image; repeat Axial 3D T1 MPRAGE as above.

Area of acquisition: A whole brain scan was used (brain and upper C-spine with inferior margin at the end of C-2 spine).

Diffusion MRI: [x] Used [ ] Not used

Parameters: Please see above.

Preprocessing

Preprocessing software	We did not perform fMRI. For our needs, we used IB Rad Tech (Imaging Biometrics, Elm Grove, WI) to do registration and dynamic susceptibility contrast (DSC) perfusion data processing.
Normalization	We did not perform fMRI. For our needs, we performed normalization for relative cerebral blood volume (rCBV) and regional cerebral blood flow (rCBF).
Normalization template	We did not perform fMRI. For our needs, we performed normalization for relative cerebral blood volume (rCBV) and regional cerebral blood flow (rCBF).
Noise and artifact removal	We did not perform fMRI; not applicable for this study.
Volume censoring	We did not perform fMRI; not applicable for this study.

Statistical modeling & inference

Model type and settings	We did not perform fMRI; not applicable for this study.
Effect(s) tested	We did not perform fMRI; not applicable for this study.

Specify type of analysis: [ ] Whole brain [ ] ROI-based [ ] Both

Statistic type for inference (See Eklund et al. 2016)	We did not perform fMRI; not applicable for our process. Please see section Treatment" on the manuscript for more details on our analysis (MRI and SPECT/CT).
Correction	We did not perform fMRI; not applicable for this study.

Models & analysis

n/a	Involved in the study
[x]	[ ] Functional and/or effective connectivity
[x]	[ ] Graph analysis
[x]	[ ] Multivariate modeling or predictive analysis
